# Chromoanagenesis Event Underlies a *de novo* Pericentric and Multiple Paracentric Inversions in a Single Chromosome Causing Coffin–Siris Syndrome

**DOI:** 10.3389/fgene.2021.708348

**Published:** 2021-08-26

**Authors:** Christopher M. Grochowski, Ana C. V. Krepischi, Jesper Eisfeldt, Haowei Du, Debora R. Bertola, Danyllo Oliveira, Silvia S. Costa, James R. Lupski, Anna Lindstrand, Claudia M. B. Carvalho

**Affiliations:** ^1^Department of Molecular and Human Genetics, Baylor College of Medicine, Houston, TX, United States; ^2^Department of Genetics and Evolutionary Biology, Human Genome and Stem Cell Research Center, Institute of Biosciences, University of São Paulo, São Paulo, Brazil; ^3^Department of Molecular Medicine and Surgery and Center for Molecular Medicine, Karolinska Institutet, Stockholm, Sweden; ^4^Science for Life Laboratory, Karolinska Institutet Science Park, Solna, Sweden; ^5^Clinical Genetics Unit, Instituto da Criança do Hospital das Clínicas, University of São Paulo, São Paulo, Brazil; ^6^Department of Pediatrics, Baylor College of Medicine, Houston, TX, United States; ^7^Human Genome Sequencing Center, Baylor College of Medicine, Houston, TX, United States; ^8^Texas Children’s Hospital, Houston, TX, United States; ^9^Department of Clinical Genetics, Karolinska University Hospital, Stockholm, Sweden; ^10^Pacific Northwest Research Institute, Seattle, WA, United States

**Keywords:** genomic inversions, structural variation, complex genomic rearrangement (CGR), chromothripsis, chromoplexy, microhomology-mediated break-induced replication (MMBIR)

## Abstract

Chromoanagenesis is a descriptive term that encompasses classes of catastrophic mutagenic processes that generate localized and complex chromosome rearrangements in both somatic and germline genomes. Herein, we describe a 5-year-old female presenting with a constellation of clinical features consistent with a clinical diagnosis of Coffin–Siris syndrome 1 (CSS1). Initial G-banded karyotyping detected a 90-Mb pericentric and a 47-Mb paracentric inversion on a single chromosome. Subsequent analysis of short-read whole-genome sequencing data and genomic optical mapping revealed additional inversions, all clustered on chromosome 6, one of them disrupting *ARID1B* for which haploinsufficiency leads to the CSS1 disease trait (MIM:135900). The aggregate structural variant data show that the resolved, the resolved derivative chromosome architecture presents four *de novo* inversions, one pericentric and three paracentric, involving six breakpoint junctions in what appears to be a shuffling of genomic material on this chromosome. Each junction was resolved to nucleotide-level resolution with mutational signatures suggestive of non-homologous end joining. The disruption of the gene *ARID1B* is shown to occur between the fourth and fifth exon of the canonical transcript with subsequent qPCR studies confirming a decrease in *ARID1B* expression in the patient versus healthy controls. Deciphering the underlying genomic architecture of chromosomal rearrangements and complex structural variants may require multiple technologies and can be critical to elucidating the molecular etiology of a patient’s clinical phenotype or resolving unsolved Mendelian disease cases.

## Introduction

Inversions are a unique class of structural variation (SV) that present at least two breakpoint junctions *in cis*. Although the majority of inversions are copy-number neutral (i.e., classical inversions), about 17% present with more complex structures accompanied with copy-number variants (CNVs) of a few bp to several kb in size (Pettersson et al., 2020). Inversion rearrangements can occur in a pericentric fashion when DNA is flipped 180° across the centromere or paracentric when the DNA inversion occurs on either the long (q) or short (p) chromosomal arm (Kaiser, 1984).

Historically, inversions were detected by cytogenetics with karyotyping; the resolution to detect such events is limited by the resolution of chromosomal G-banding (approximately 5–10 Mb). Routine genomic testing including array comparative genomic hybridization (aCGH) and exome sequencing (ES) will not detect most inversion events given that they are typically: (1) copy-number neutral and (2) usually do not have breakpoints within the coding regions targeted by ES (Posey, 2019; Lupski et al., 2020). The advent of short-read whole-genome sequencing (WGS) enabled detection of inversion events, though the rate of false-positives (Vicente-Salvador et al., 2017) as well as false-negatives is very high, the latter due to lack of detection of inversions with breakpoints within repetitive regions (Chaisson et al., 2019). Recently, long-read DNA sequencing, e.g., Oxford Nanopore and PacBio, and genomic optical mapping, e.g., Bionano, as well as Strand-seq have resulted in increased sensitivity of inversion detection as they allow accurate genotype and phasing of events with multiple breakpoints junctions *in cis*, including those mapping to genomic repeats (Ebert et al., 2021).

In the constitutional genome, inversions have been shown to be formed through three different molecular mechanisms sometimes acting in concert (Pettersson et al., 2020). Non-allelic homologous recombination (NAHR) is one driver of inversion formation when breakpoints are found to be part of a pair of inverted genomic segments sharing sequence homology (Flores et al., 2007; Kidd et al., 2008). Micromology-mediated end joining (MMEJ) or non-homologous end joining (NHEJ) are the most likely mechanisms generating inversions with breakpoints presenting very little or no microhomology (Pettersson et al., 2020). For copy-number associated inversions observed in complex genomic rearrangements (CGRs), replicative mechanisms, such as microhomology-mediated break-induced replication (MMBIR) play a role in the inversion formation process (Lee et al., 2007; Carvalho et al., 2011; Beck et al., 2015; Gu et al., 2015; Pettersson et al., 2020). As inversions can be formed by one or more molecular mechanisms, each individual case must be resolved to nucleotide-level resolution to infer the molecular mutational mechanism(s) that may have been involved.

Inversion formation can cause gene disruptions and amplifications and have been implicated in the evolution of novel genes and “exonization” of gene structures (Lakich et al., 1993; Carvalho et al., 2011; Zuccherato et al., 2016). Gene interrupting inversions are implicated in some genomic disorders most notably an inversion physically separating parts of the *F8* gene, the most common cause of severe hemophilia A (Lakich et al., 1993). The pathogenetic consequence of this type of structural variant may result from a breakpoint occurring within the exon of a gene or in an intragenic fashion between exons (Feuk, 2010); the end result is a gene split apart disrupting its function (Lakich et al., 1993). More cryptically, inversions may disrupt enhancer or topologically associated domains surrounding a gene, causing no change in the gene itself but leading to a pathogenic consequence through change in gene expression, a potential position effect, or other perturbations of gene regulation (Lupianez et al., 2015; Kraft et al., 2019; Sanchez-Gaya et al., 2020).

Herein, we present a patient with Coffin–Siris syndrome 1 (CSS1) and multiple inversions affecting a single chromosome. Complex structural variants have been shown to present a challenge for detection as well as molecular and genomic characterization partly due to the inability to properly phase detected variants, as well as subsequent clinical interpretation of potential contribution of variant effects to observed clinical phenotype(s) (Grochowski et al., 2018; Eisfeldt et al., 2020; Plesser Duvdevani et al., 2020). To experimentally dissect the genomic architecture of the rearranged chromosome 6 of this patient, and to explore whether genes involved in the rearrangement contributed to the observed clinical traits, we employed several technologies including karyotyping (G-banding), fluorescence *in situ* hybridization (FISH), quantitative PCR (qPCR), aCGH, WGS, and genomic optical mapping in this study. The convergence of experimental approaches allowed for DNA base-pair resolution of the genomic inversion rearrangements and revealed that an inversion caused disruption of the gene *ARID1B*, explaining the clinical phenotype in this patient. Furthermore, our studies revealed a rare chromoanagenesis event constituted by multiple copy-number neutral inversions.

## Materials and Methods

### Patient Enrollment

The affected proband and unaffected sister, mother, and father were evaluated and characterized at the University of São Paulo (Protocol 2.589.398). The trio (proband, mother, and father) were subsequently enrolled under a protocol approved by the institutional review board at Baylor College of Medicine (IRB #: H-29697). Genomic DNA was extracted from peripheral blood using standard protocols.

### Conventional Karyotyping and Cytogenomic Studies

GTG-banding karyotypes from cultured peripheral blood lymphocytes were obtained following standard protocols (Supplementary Figure 1). FISH on metaphase chromosomes was implemented using bacterial artificial chromosome (BAC) DNAs from the 1-Mb clone set^1^ mapped to the long arm of chromosome 6 (RP11-506N21, RP3-336G18, and RP11-266C7). Metaphase spreads were analyzed using a Zeiss fluorescence microscope and processed using ISIS software (MetaSystem). At least 20 metaphase spreads from the patient and her parents were analyzed.

### Array Comparative Genomic Hybridization (aCGH)

Initial aCGH analyses were performed using a 180K genome-wide Agilent array. A subsequent custom 180K Agilent high-resolution array was designed to interrogate both the long and short arm of chromosome 6 (AMADID#: 086000) using the Agilent e-array website^2^ (Santa Clara, CA, United States) with a median probe spacing of 857 bp maximally spaced across the entire chromosome 6. Array experiments were conducted following protocols set forth by Agilent in relation to hybridization and labeling with minor modifications (Carvalho et al., 2009; Supplementary Figure 2A).

### Short-Read WGS

Short-read WGS was performed using Illumina 30× PCR-free paired-end (PE) DNA sequencing (Hofmeister et al., 2018) at the National Genomics Infrastructure (NGI), in Stockholm, Sweden. All data obtained were processed using NGI-piper and analysis for structural variants was performed using the FindSV pipeline^3^ (Supplementary Figure 2B). FindSV combines CNVnator V.0.3.2 (Abyzov et al., 2011) and TIDDIT V.1.1.4 (Eisfeldt et al., 2017) and produces a single variant calling format (VCF) file, subsequently annotated by variant effect predictor (VEP) and filtered based on the VCF file quality (McLaren et al., 2010). Lastly, the VCF file is sorted based on a local structural variant frequency database consisting of 351 personal genome samples of well-characterized healthy and affected individuals, and the SV of interest was identified based on the VEP annotation and variant frequency. Manual inspection and identification of split reads was performed using the Integrative Genomics Viewer (IGV)^4^ (Robinson et al., 2011). Exact genomic map positions of breakpoints, at the nucleotide level, could then be determined by alignment of split reads to the Hg19/GRCh37 reference genome using the BLAST-like alignment tool (BLAT)^5^ (Kent, 2002). Single-nucleotide variants (SNVs) overlapping the inversions were extracted using Tabix (Li, 2011). SNVs were called as previously described (Pettersson et al., 2020), and the resulting call sets were filtered for *de novo* SNV using BCFtools (Li et al., 2009). *De novo* and inherited SNV and indels were filtered and annotated based on the mutation identification pipeline (MIP) clinical workflow and sorted based on allele frequency, variant consequence, and CADD score.

### qPCR Gene Expression Analysis

Total mRNA was extracted from peripheral blood using the RNeasy mini kit (Qiagen) following the manufacturer’s instructions. After evaluating RNA integrity and concentration with a NanoDrop spectrophotometer (Thermo Fisher Scientific), 1 μg of RNA was used for cDNA synthesis with a SuperScript III First-Strand Synthesis System and oligo-dT primers (Thermo Fisher Scientific). Real-Time qPCR (RT-qPCR) experiments were performed in triplicate in a 7500 Fast Real-Time PCR System, using SYBR Green PCR Master Mix (Thermo Fisher Scientific). Primers for *ARID1B* were guided and designed using Primer3 software (forward: 5′ GGCCGTCCCGGAGTTTAATAA 3′ and reverse: 5′ CGGAGTGCATCATCCCCAT 3′), with efficiency being evaluated by serial cDNA dilutions This primer set targets a region of exon 1 in *ARID1B* of the transcript NM_001374820.1. The endogenous control *GAPDH* was used as a normalizing factor for each sample (primers: forward: 5′ GCATCCTGGGCTACACTG 3′ and reverse: 5′ CCACCACCCTGTTGCTGTA 3′). Unpaired *t*-test was applied in the statistical analyses, through SPSS V22 software.

### Genomic Optical Mapping

High molecular weight (HMW) genomic DNA for use in genomic optical mapping was extracted by Histogenetics (Ossining, NY, United States) from whole blood using the Bionano Prep Blood and Cell Culture DNA Isolation Kit (Bionano Genomics). Subsequent DNA quantity and size were confirmed using a Qubit dsDNA BR Assay Kit. A total of 0.75 μg of HMW DNA was then labeled using the Bionano Prep direct label and stain (DLS) method (Bionano Genomics) and loaded onto a flow cell to run on the Saphyr optical mapping system (Bionano Genomics) (Supplementary Figure 2C). Approximately 230–370 Gb of data were generated per run. Raw optical mapping molecules in the form of BNX files generated from a diploid genome were parsed through a preliminary bioinformatic pipeline that filtered out molecules less than 150 kb in size and with less than nine motifs per molecule to generate a *de novo* assembly of the genome maps. Data were then aligned to an *in silico* reference genome (Hg38/GRCh38) using the Bionano Solve v3.5 RefAligner module. Structural variant calls were generated through comparison of the reference genome using a custom Bionano SV caller. Manual inspection of proposed breakpoint junctions was then visualized in the Bionano Access software program v1.5.1.

### Bionano SV Analysis

Optical mapping was run on the Saphyr platform^6^ at Bionano Genomics (San Diego, CA, United States). The optical maps were analyzed using the Bionano-solve pipeline^7^. Briefly, the maps were detected using AutoDetect, and assembled using the *de novo* assembly package AssembleMolecules. The resulting consensus maps were aligned to Hg19/GRCh37 using the Bionano RefAligner. Lastly, the variants of interest were visualized using Bionano Access, and the resulting smap files were converted to VCF using a custom version of the smap2vcf script^8^. *De novo* SVs were discovered by merging these VCF files into a single trio-VCF. The SVs were merged using SVDB v2.3.0, and variants unique to the proband were discovered using the GNU grep tool (Eisfeldt et al., 2017).

### *De novo* GATK Filtering

Individual germline SNVs and indels were called using GATK (v.4.1.3) (McKenna et al., 2010). Of note, ‘‘-GVCF’’ option was used for GATK haplotypecaller, which outputs a gVCF file that includes reference or variant information for all loci. The gVCF files for a family were combined and the proband’s genotype was recalibrated based on parental genotype per Mendel’s laws of allele transmission. Using recalibrated posterior genotype probabilities, possible *de novo* mutations were tagged. All possible *de novo* variants were filtered by an in-house developed software called DNM (*de novo* mutation)-Finder^9^ that combines GATK and xAtlas (Eldomery et al., 2017).

### Chromosome Rearrangement Simulation

A Monte Carlo simulation to test the likelihood of chromosomal breakpoints occurring in specific locations was designed to mirror the rearrangement observed in this patient. Briefly, the base pairs encompassing chromosome 6 (chr6:1-171,115,067) were broken into seven segments with only the first and last segment being positionally static. The remaining five segments could be randomly reshuffled with a 50% chance of inverting. The breakpoint positions of these segments were randomly and uniformly selected across chromosome 6. The simulation was run 10,000 times to statistically test for significance of clustering or enrichment of breakpoints within protein-coding genes on chromosome 6 (according to ENSEMBL release 87). The clustering of the breakpoints was assessed by computing the average distance between breakpoints; a simulated rearrangement was considered more clustered if its average breakpoint distance was smaller than the average breakpoint distance observed in the index patient. The enrichment of protein-coding genes was assessed by counting the number of breakpoint junctions carrying fusions of protein-coding genes. The scripts needed for extracting the protein coding genes and running the simulation are available on git-hub^10^.

### Breakpoint PCR Sequencing

The precise location of each breakpoint junction identified in the WGS data were determined and visualized with IGV. For each position, the relative strand orientation (i.e., polarity), and the genomic map position on the haploid reference human genome, of the junction was identified. Primers were designed upstream and downstream of the identified junction and PCR amplification was performed using the HotStarTaq (Qiagen) polymerase with standard conditions. Sanger-sequencing was performed at the Baylor College of Medicine Sequencing Core, and the results were visualized using the Sequencher software suite (Genecodes).

## Results

### Pericentric and Paracentric Inversions on Chromosome 6

**FIGURE 1:**
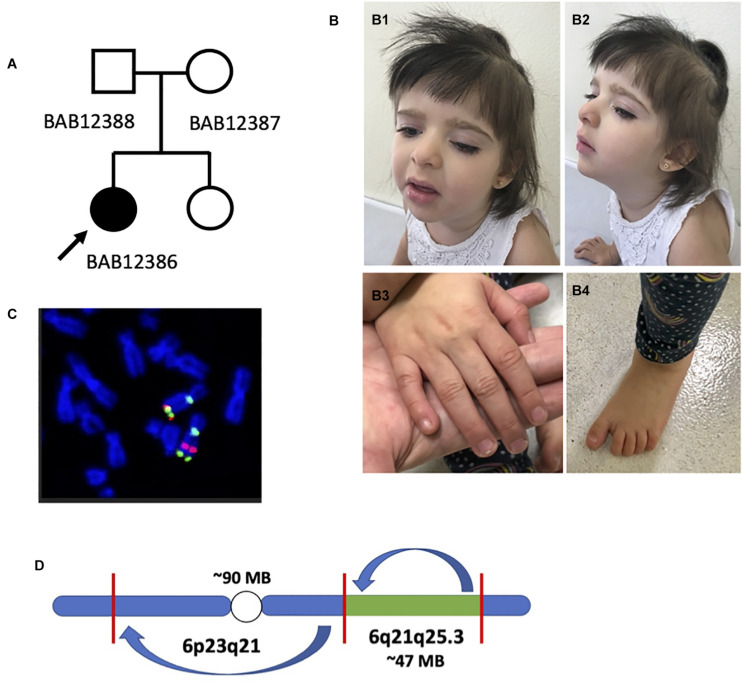
Preliminary analysis of proband and chromosome 6 rearrangement. **(A)** Pedigree structure with the father (BAB12388), mother (BAB12387), and proband (BAB12386) as well as an unaffected sister (not enrolled). **(B)** Female proband (BAB12386) highlighting mildly dysmorphic facies and typical hand features. **(B1,B2)** Frontal and lateral view of the proband at the age of 4 years showing thick hair with sparseness in the temporal region, bushy eyebrows and long eyelashes, left palpebral ptosis, and full lips with eversion of the lower lip. **(B3,B4)** Right hand and foot depicting normal nails and increased distance between the hallux and second toe (sandal gap sign). **(C)** Fluorescence *in situ* hybridization (FISH) analysis confirming apparent pericentric and paracentric inversions present on chromosome 6 as first detected by karyotyping analysis. **(D)** Initially proposed chromosome 6 structure with a ∼90-Mb and ∼47-Mb inversion both present on chromosome 6.

The 5-year-old female proband is the first child born to non-consanguineous healthy parents (29-year-old mother and 30-year-old father) at 39 weeks gestational age, i.e., full term, by cesarean section, after an uneventful pregnancy (Figure 1A). She has one younger sister with no history of physical or developmental abnormalities. Her birth weight was 2,345 g (<10th centile), her length was 44 cm (<10th centile), and her occipital frontal circumference (OFC) was 33.5 (50th centile). Apgar scores were 9 and 9 at 1 and 5 min, respectively. She was sent home after 3 days in the hospital. There were no major pregnancy or birth complications or any birth defects recognized on newborn examination.

The mother first noticed poor suck with hypotonia during the first week of life, evolving with poor weight gain and developmental delay: she sat unsupported at 9 months of age and crawled at 18 months. At the age of 4 years, she was not able to walk unassisted and she had not developed speech. She was evaluated by a neurologist in the first months of life and started physical therapy at 5 months of age with a treatment goal to improve her motor skills. At that time, cranial computed tomography scans and screening for inborn errors of metabolism were both normal and she never presented with any seizure disorder. An ophthalmologic evaluation disclosed strabismus, which required surgical correction at the age of 1 year and 10 months though she developed a left ptosis after the procedure.

Cardiologic evaluation disclosed an atrial septal defect (ASD), ostium secundum type, of 10 mm at 7 months of age. Further complementary exams, including audiological evaluation, abdominal ultrasound, and spine x-rays, were normal. She was evaluated by a clinical geneticist at 14 months of age and genetic tests disclosed a G-banded karyotype showing two rearrangements [46,XX, der(6)inv(6)(p23q21)inv(6)(q21q25.3)] and a normal chromosomal microarray, indicating balanced chromosomal rearrangements. Subsequent G-banded karyotyping of her mother did not indicate presence of the rearrangement. The proband also manifested premature thelarche and has been followed by an endocrinologist, with normal hormonal profile.

Physical examination at the age of 3 years showed a weight of 11.760 g (5th centile), height of 89 cm (10th centile), and OFC of 47 cm (2nd to 50th centile); there was thick hair, with sparseness in the parietal region. Facial dysmorphology was notable for bushy eyebrows, long eyelashes, and ocular asymmetry with left palpebral ptosis (Figure 1B). There was a long and prominent columella, widely spaced teeth, full lips with everted lower lip, and retrognathia. Palpable breast tissue was noted. Extremities were notable for hypertrichosis in upper limbs and dorsum; finger pads, single transverse palmar creases, and normal nails; and flat feet, with sandal gap deformity (Figure 1B and Supplementary Figure 3). Genitourinary exam showed hypoplastic labia minora. The diagnosis of Coffin–Siris syndrome was raised based on the clinical findings presented by the proband.

To further characterize the chromosomal abnormality, conventional clinical cytogenetics karyotyping using G-banding was repeated in the child and performed in both parents. These studies revealed a *de novo* apparently balanced rearrangement on chromosome 6 involving one pericentric and one paracentric inversion: 46,XX, der(6)inv(6)(p23q21)inv(6)(q21q25.3) (Supplementary Figures 1, 4). Dual-color fluorophore FISH confirmed the two inversions and allowed mapping of one of the cytogenetic breakpoints. In the rearranged chromosome 6, the pericentromeric 6q genomic probe BAC RP11-506N21 (green) was detected on the short arm, confirming the pericentric inversion (Figure 1C). Regarding the two 6q25.3 probes, only the sequence RP3-336G18 (red) has moved to a location at 6q more proximal to the centromere; this result confirmed the paracentric inversion, mapping the breakpoint at 6q25.3 to a genomic segment of 1.2 Mb delimited by the clones RP3-336G18 and RP11-266C7 (Figure 1C and Supplementary Figure 5), which contains *ARID1B*, a potential candidate gene for the proband’s proposed clinical diagnosis. Given this information, the original proposed architecture of chromosome 6 involved an approximately 90-Mb pericentric inversion and 47-Mb paracentric inversion based on a human haploid reference genome map (Figure 1D).

### Evidence for Additional Chromosome 6 Inversions

We performed Illumina 30X PCR-free paired-end (PE) WGS on genomic DNA samples from the proband and parents to identify *de novo* mutational events that might be associated with the apparent sporadic disease. Subsequently, the TIDDIT structural variant caller parsed *de novo* SVs genome-wide (Eisfeldt et al., 2017). Analysis of *de novo* SVs affecting chromosome 6 confirmed the presence of the paracentric and pericentric inversions observed by cytogenetic and cytogenomic studies and revealed three additional breakpoints localized on the long arm at 6q25.3 corresponding to a potential third inversion event not observed previously (Supplementary Table 1). The three novel junctions are constituted of ∼1-Mb fragments mapping telomeric to the 46.21-Mb pericentric inversion on 6q. Two out of six structural variants were called as “blunt-end” by the algorithm caller and the remaining four involved in this chromosome were called as an inversion. All regions were manually inspected in IGV (Supplementary Figure 6) and the break disrupting the gene *ARID1B* was confirmed (Chr6:157,240,695; Hg19/GRCh37). To determine if the inversions generated were accompanied by CNVs, we performed a custom high-resolution aCGH targeting chromosome 6. No *de novo* CNVs were detected in the proband or parent genome, confirming that, indeed, these inferred SVs were copy-number neutral events affecting only chromosome 6 (Supplementary Figures 7, 8). Genome-wide optical mapping and SV analysis from WGS data showed no additional potentially pathogenic variation.

GATK analysis showed approximately 61 *de novo* SNVs and indels detected genome-wide with no enrichment around the identified breakpoint junctions on chromosome 6. No other potentially pathogenic variants were detected after filtering and annotation for *de novo* or inherited variation.

### Genomic Rearrangement Architecture and Recombinant Junction Sequences

**FIGURE 2:**
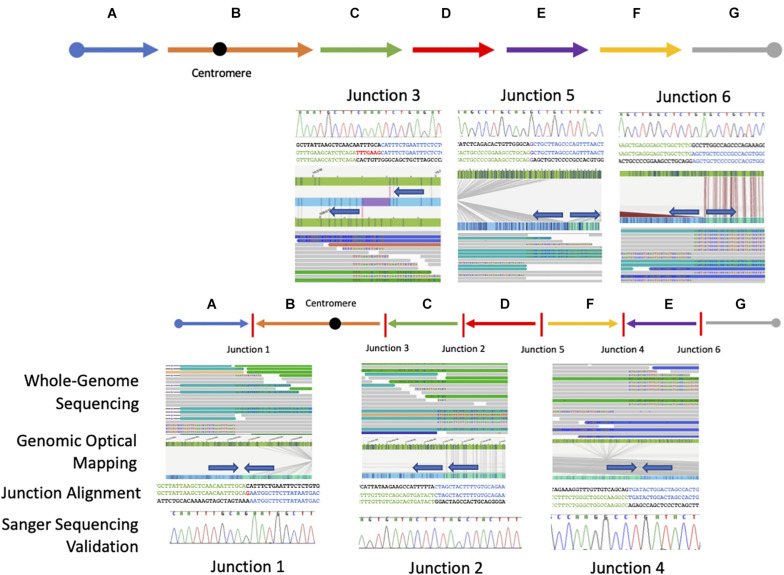
Resolved breakpoint junctions as visualized by multiple genomic technologies. The final resolved chromosome 6 structure showing each genomic fragment orientation and the six breakpoint junctions as visualized through each technology applied including whole-genome sequencing, genomic optical mapping, breakpoint-junction alignment, and final Sanger validation. The colored arrows at the top represent the reference orientation of each genomic fragment. The arrow orientation in the middle represents the orientation of each genomic fragment in this patient with respect to reference.

Starting from the distal breakpoint position on the p arm, the pericentric inversion is highlighted as segment B (Figure 2). The genome map position then connects to segment C on the q arm, in an inverted orientation, which then connects to segment D also in an inverted orientation. Segments E and F are in opposite positions relative to each other with segment F connecting to segment D in the reference orientation and segment E connecting to segment F in an inverted orientation.

**FIGURE 3:**
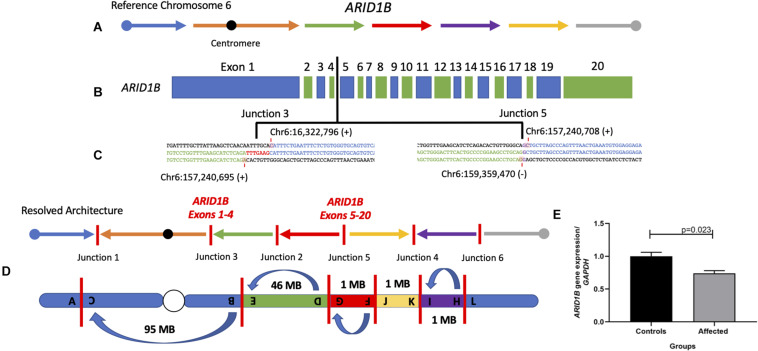
Final chromosome 6 resolved architecture revealed disruption of the gene *ARID1B*. **(A)** Structure of chromosome 6 displaying the reference orientation of each genomic fragment as represented by arrows moving from left to right with the centromere positioned as a black circle. **(B)** Coding structure of the gene *ARID1B* (NM_001374820.1). Vertical black line indicates the inversion break that disrupted the gene between the fourth and fifth exons. **(C)** Breakpoint sequence alignments of junctions 3 and 5 indicate the nucleotide positions disrupted within *ARID1B*. **(D)** Final resolved structure of chromosome 6 showing six breakpoint junctions with one pericentric inversion and three paracentric inversions on the q arm. **(E)** qPCR analysis of *ARID1B* mRNA in patient compared to three controls reveal significant expression reduction (∼30%) in peripheral blood.

Sequence alignments showed that junctions 2, 4, and 6 have a blunt breakpoint junction, whereas junction 5 shows a one base pair of microhomology (G) and junction 1 had a one nucleotide insertion of a “G” (Figure 2 and Supplementary Figure 9). Finally, junction 3 showed an apparent seven-nucleotide templated insertion of “TTTGAAG” likely originating from 9 bp upstream of the proximal strand. The relatively simple features (blunt fusion, microhomology, and small insertions) of the breakpoint junctions and copy-number neutral state of the rearrangement allows inference of a possible DNA NHEJ mechanism as a likely mechanism for generation of formation for this chromosomal aberration. Together, the proposed architecture using the orientation and directionality for each genomic fragment from the nucleotide-level junction alignments and the *de novo* mutation event in sporadic disease implicates this complex rearrangement as clinically relevant for this proband (Figure 3).

### Genomic Optical Mapping Supports Genomic Orientation and Architecture

To orthogonally investigate this CGR and proposed genomic architecture of the SV haplotype involving chromosome 6, we performed DLS genomic optical mapping. After the identification and sequence alignment of the breakpoint junctions were obtained, we interrogated the genomic optical mapping data at those nucleotide positions. Although the inversion events were too large (>1 Mb) to capture on a single DNA molecule, *de novo* assembly of the patient’s personal genome allowed consensus contigs to span the region upstream and downstream of each breakpoint position. Each junction orientation and connection identified in the WGS data were validated in optical genome mapping by visualizing directionality or polarity of sequence motifs in an inverted or direct recombinant join-point connection (Figure 2 and Supplementary Figures 10–16). The molecules spanning the breakpoint junctions were visually inspected, and scrutinized, to parsimoniously map and positionally assign each genomic fragment visualized with optical sequence motifs consistent with the genomic fragment connection.

### Inversion Results in Measurable Reduction in Gene Dosage Expression

Importantly, *ARID1B* is disrupted in one location, between the fourth and fifth exons of the transcript NM_001374820.1, and generated breakpoint junction 3 (chr6:157,240,695; Hg19/GRCh37) and junction 5 (chr6:157,240,708), *in cis* (Figure 3 and Supplementary Figure 17). Disruption of the gene *ARID1B* through loss-of-function (LoF) variants has been shown to cause CSS1 (Hoyer et al., 2012; Santen et al., 2012, 2013). The expression levels of *ARID1B* were assayed, with its relative expression compared to three normal controls, to determine if the inversion splitting the gene disrupted its expression in peripheral blood. The levels were significantly (*p* = 0.023, *n* = 3) reduced 30% when compared to normal control samples against the *GAPDH* housekeeping gene.

## Discussion

Herein, we present a CGR involving chromosome 6 that disrupts the gene *ARID1B* causing CSS1. The initial karyotyping and FISH analysis, i.e., single cell genomics, indicated one pericentric and one paracentric inversion of chromosome 6. Higher-resolution genomic approaches including WGS and genomic optical mapping uncovered a more complex chromosomal aberration with one (∼95 Mb) pericentric inversion and three additional paracentric inversions (∼46, ∼1, and ∼1 Mb), all of which are localized to a single chromosome 6 in a *de novo* copy-number neutral mutational event. A combination of experimental methods and genomic approaches resolved the genomic structure of the derivative chromosome 6.

Coffin–Siris syndrome 1 is a clinically and genetically heterogeneous disorder with the most frequent clinically observed findings being developmental delay, coarse facial features, feeding difficulties, frequent infections, and hypoplastic or absent fingernail on the fifth digit (Fleck et al., 2001; Santen et al., 2013). In 2012, both heterozygous deletions and point mutations in the switch/sucrose non-fermentable SWI/SNF-like chromatin remodeling complex gene *ARID1B* were reported to cause CSS1 in a monoallelic, autosomal dominant trait inheritance, Mendelian model (Hoyer et al., 2012; Santen et al., 2012). Although several other genes encoding proteins in the SWI/SNF-like BAF complex including *ARID1A*, *SMARCA2*, *SMARCA4*, *SMARCB1*, and *SMARCE1* have also been shown to cause the Coffin–Siris syndrome phenotype (Santen et al., 2013), and/or a CSS-like phenotype, *ARID1B* is recognized as one of the most frequently mutated genes causing intellectual disability (Hoyer et al., 2012; Santen et al., 2014; Yang et al., 2014; Liu et al., 2019).

The proband described herein (BAB12386) presented with many of the well-characterized phenotypic features of the disease trait including developmental delay, typical craniofacial dysmorphisms, hypotonia with feeding difficulties, hypertrichosis and sparse scalp hair, and premature thelarche, the latter a rare finding reported in CSS1 (Vergano and Deardorff, 2014; Figure 1 and Supplementary Figure 3). Notably absent is the hypoplastic fifth finger or toenail, which appears normal in the present patient (Figure 1B and Supplementary Figure 3), but can be observed in 81–95% of patients with clinically diagnosed CSS1 (Fleck et al., 2001; Santen et al., 2014). We cannot rule out that hypoplastic phalanges are not present in our patient, since no hand x-ray studies were performed.

There were other genes involved in the rearrangement including *ATXN1*, *CDK19*, and *SYNJ2* (Supplementary Figure 18). In mice, deletions of *ATXN1* have been shown to cause mild learning defects without neurodegeneration (Lu et al., 2017). Recently, missense variants in *CDK19* have been shown to cause developmental and epileptic encephalopathy (MIM:618916), though partial gene deletions have been found in healthy individuals suggesting that haploinsufficiency of *CDK19* may not be clinically relevant (Wong et al., 2007; Chung et al., 2020). *SYNJ2* has been shown to be involved in the formation of cell membrane structures though the gene has not been directly linked to a human disease state (Chuang et al., 2004). Therefore, disruption of *ARID1B* is a plausible explanation from the genomic and clinical points of view. Nevertheless, we cannot completely rule out a blended phenotype (Posey et al., 2017) that may occur due to the disruptions of *ATXN1* as well as *CDK19* or the contributory role of other gene loci and genetic variation potentially conferring position effects due to the complex reordered genome and chromosome structure present on chromosome 6.

Structural variation, including deletions, intragenic duplications, and translocations leading to disruptions of *ARID1B*, has been previously reported (Halgren et al., 2012; Seabra et al., 2017). The disruption of *ARID1B* that drives this patient’s phenotype appears to have occurred as the result of a balanced inversion event translocating the proximal and distal *ARID1B* transcripts to two different genomic locations. This genomic rearrangement resulted in an observed 30% reduction of *ARID1B* specific mRNA dosage or expression as observed by RT-PCR in diploid cells (Figure 3E). It is intriguing that the levels of *ARID1B* expression in blood is reduced by 30% rather than the expected 50%. We speculate that there is higher expression of the wild-type (WT) allele in blood, perhaps due to compensation or that the qPCR experiment performed is measuring both the WT and truncated transcripts, the latter not fully degraded by nonsense-mediated decay as would be expected. Interestingly, similar ∼30% decreased mRNA expression has been detected in another patient with SV affecting *ARID1B* also clinically diagnosed with CSS1 (Halgren et al., 2012; Seabra et al., 2017). The qPCR primer sets used to assay *ARD1B* in our study as well as Seabra et al. (2017) target three out of four transcripts of the gene including the canonical transcript.

The complex genomic structure and mutational junction signatures appear to have been formed by an NHEJ mechanism generating this highly reordered chromosome. Chromoanagenesis, i.e., chromosome rebirth, encompasses the phenomena of extensive rearrangement occurring in a single burst (including chromothripsis, chromoanasynthesis, and chromoplexy), generating localized complex chromosome rearrangements identified in both somatic and germline genomes (Holland and Cleveland, 2012; Ly and Cleveland, 2017). Although this type of aberration complies with some aspects of chromothripsis, including the involvement of one chromosome and six breakpoints with genomic fragment shuffling in a balanced manner (Kloosterman et al., 2011, 2012; Maher and Wilson, 2012), the fact that the breakpoints are not clustered and appear to occur within transcriptionally active areas (four out of six breakpoints occur within genes) is also in line with a chromoplexy-type event (Shen, 2013; Redin et al., 2017). Although chromothripsis and chromoplexy were first characterized in cancer genomes, the same “mutagenic phenomenon” has been shown to underlie Mendelian diseases and genomic disorders by disruption of genes through truncating breakpoints (haploinsufficiency), by the generation of fusion genes (ectopic expression), or other position effects (Maher and Wilson, 2012; Baca et al., 2013; Redin et al., 2017; Plesser Duvdevani et al., 2020). This process may occur in a random order of DNA fusion but interestingly in this present case, almost all the inversion events happen sequentially from one another in a potential “chained” fashion rather than a single “pulverizing” event which is more suggestive of chromoplexy (chained rearrangements) over chromothripsis (a single catastrophic event occurring).

To test the likelihood that this rearrangement is formed through a chromoplexy- versus chromothripsis-type mechanism, we performed a simulation to test for either an enrichment of breakpoints occurring within protein coding genes (which would support chromoplexy) or a clustering of breakpoints on the chromosome (which would support chromothripsis). After 10,000 simulations, we observed neither a significant enrichment of breakpoints within protein coding genes (*p*-value of 0.112) nor a denser clustering of breakpoints than would be expected by chance (*p*-value of 0.758), suggesting an expanded understanding of mutation events that appear to fall under the chromoanagenesis definition.

In summary, resolving the CGR affecting chromosome 6 required the use of multiple technologies to elucidate the structure of a derivative chromosome constituted by multiple copy-number neutral events. Resolving this genomic puzzle was key to identify the underlying molecular cause of the clinical traits in this patient. Moreover, the identification of several *de novo* inversions on a single chromosome, generated through a chromothriptic-like mutational event, suggests that such mutational process may lead to hidden complexities in seemingly “simple” structural variants. As we continue to refine and improve our ability to resolve inversions and other complex structural variants, “unsolved” Mendelian diseases should be investigated by applying new and developing genomic methodologies that allow phasing multiple breakpoint junctions *in cis* (Liu et al., 2019; Plesser Duvdevani et al., 2020).

## Data Availability Statement

Microarray data generated in this study are available through GEO under the accession number GSE180423. BAM files for the proband indicating the specified structural variants are deposited in the Sequence Read Archive (SRA), accession number PRJNA748013.

## Ethics Statement

The studies involving human participants were reviewed and approved by Baylor College of Medicine (IRB #: H-29697). Written informed consent to participate in this study was provided by the participants’ legal guardian/next of kin. Written informed consent was obtained from the individual(s), and minor(s)’ legal guardian/next of kin, for the publication of any potentially identifiable images or data included in this article.

## Author Contributions

CMG performed the laboratory work, analyzed and interpreted the data, and wrote the manuscript. JE and HD performed the bioinformatic analysis. ACVK, DRB, DO, and SSC provided patient samples, clinical information of patients, and/or analysis and interpretation of data. JRL and AL performed data interpretation and critical review of the manuscript. CMBC conceptualized the study, analyzed and interpreted the data, and is a major contributor in writing the manuscript. All authors have read, edited, and approved the final manuscript.

## Conflict of Interest

Baylor College of Medicine (BCM) and Miraca Holdings have formed a joint venture with shared ownership and governance of the Baylor Genetics (BG), which performs clinical microarray analysis and other genomic studies (ES and WGS) for patient/family care. JRL serves on the Scientific Advisory Board of the BG. JRL has stock ownership in 23andMe, is a paid consultant for Regeneron Pharmaceuticals, and is a co-inventor on multiple United States and European patents related to molecular diagnostics for inherited neuropathies, eye diseases, and bacterial genomic fingerprinting. The remaining authors declare that the research was conducted in the absence of any commercial or financial relationships that could be construed as a potential conflict of interest.

## Publisher’s Note

All claims expressed in this article are solely those of the authors and do not necessarily represent those of their affiliated organizations, or those of the publisher, the editors and the reviewers. Any product that may be evaluated in this article, or claim that may be made by its manufacturer, is not guaranteed or endorsed by the publisher.

## Footnotes

1
http://www.ensembl.org/

2
http://earray.chem.agilent.com/earray/

3
https://github.com/J35P312/FindSV

4
http://software.broadinstitute.org/software/igv/

5
https://genome.ucsc.edu/cgi-bin/hgBlat

6
https://bionanogenomics.com/support-page/saphyr-system

7
https://bionanogenomics.com/support-page/bionano-solve

8
https://github.com/J35P312/smap2vcf

9
https://github.com/BCM-Lupskilab/DNM-Finder

10
https://github.com/J35P312/MonteSV

## Supplementary Material

Supplementary Figure 1 Proband (BAB12386) full karyotype highlighting chromosome 6 within the red outlined box [46,XX, der(6)inv(6)(p23q21)inv(6)(q21q25.3)].

Supplementary Figure 2 After initial karyotyping and fluorescent *in situ* hybridization a combination of methods. **(A)** Trio custom high-resolution array comparative genomic hybridization with average probe spacing of 1 probe per 857 bp spanning chromosome 6. **(B)** Illumina 30X PCR-free paired-end (PE) trio whole-genome sequencing and **(C)** Direct Label and Stain (DLS) trio genomic optical mapping was performed on the Bionano Saphyr system.

Supplementary Figure 3 **(A)** Proband (BAB12386) clinical photos denoting thick hair, with sparseness in parietal region, bushy eyebrows, long eyelashes, ocular asymmetry with left palpebral ptosis, long and prominent columella, full lips with everted lower lip, retrognathia; **(B)** hypertrichosis in upper limbs and dorsum; **(C)** finger pads, single transverse palmar creases, normal nails; flat feet, with sandal gap deformity.

Supplementary Figure 4 Original karyotyping analysis using G-banding showing a *de novo* apparently balanced rearrangement on chromosome 6 involving one pericentric and one paracentric inversions: 46,XX, der(6)inv(6)(p23q21)inv(6)(q21q25.3).

Supplementary Figure 5 Dual-color fluorophore FISH confirmed the two inversion events. The pericentromeric 6q genomic probe, bacterial artificial chromosome (BAC) RP11-506N21 (green) was detected on the short arm, confirming the pericentric inversion. The RP3-336G18 probe (red) has moved to a location at 6q more proximal to the centromere; this result confirmed the paracentric inversion, mapping the breakpoint at 6q25.3 to a genomic segment of 1.2 Mb delimited by the clones RP3-336G18 and RP11-266C7.

Supplementary Figure 6 All breakpoint regions were manually inspected in the integrative genomics viewer (IGV) showing soft-clipped reads flaking each region.

Supplementary Figure 7 **(A)** A high-resolution aCGH targeting the long and short arm of chromosome 6 with a median probe spacing of 857 bp across the chromosome was performed in the proband (BAB12386) as well as the mother (BAB12387) and father (BAB12388). No CNVs were detected across the chromosome or **(B)** surrounding the gene *ARID1B*.

Supplementary Figure 8 The regions surrounding each breakpoint were scrutinized in the aCGH for the proband, mother and father. No small CNVs were detected for any of their 6 breakpoint regions.

Supplementary Figure 9 Nucleotide-level resolution of all 6 breakpoint junctions are shown with the nucleotide position (Hg19/GRCh37) from each side as well as the directionality of the sequence (±) forming the junction.

Supplementary Figure 10 **(A)** Representation of junction one showing break one in a positive orientation connecting to break 3 in a negative orientation as expected for an inversion event. **(B)** Genomic optical mapping data shows the positive and negative orientation as well as the genomic coordinates of the breakpoint connecting in an inverted manner.

Supplementary Figure 11 Junction 1 optical mapping data showing single molecule support of the breakpoint junction architecture. A single molecule spanning the junction with a length of approximately 429 kb is highlighted by the red boxes.

Supplementary Figure 12 Genomic optical mapping data representing junction 2 with break 4 (Chr6:111,024,035) and break 7 (Chr6:158,471,524) connecting in tandem.

Supplementary Figure 13 **(A)** Genomic optical mapping data for junction 3 showing the point of connection for break 2 (Chr6:157,240,695) in tandem. **(B)** The connection between *ARID1B* (purple) to *ATXN1* (green).

Supplementary Figure 14 Genomic optical mapping data for junction 4showing the connection of Chr6:158,471,518 and Chr6:160,535,951 in an inverted orientation.

Supplementary Figure 15 Genomic optical mapping data with single molecule visualization for junction 5. The connection at Chr6:158,471,518 is which is represented by the purple arrow in a tail-to-tail orientation with Chr6:157,240,708 which is represented by a red arrow.

Supplementary Figure 16 Genomic optical mapping data showing junction 6 with the connection of Chr6:159,359,468 which is represented by a yellow arrow fused to Chr6:160,535,951 represented by a gray arrow connected in a tail-to-tail orientation.

Supplementary Figure 17 Graphical representation of the exons forming *ARID1B* as well as the position of the inversion breakpoint disrupting the gene as denoted by the red vertical line between the 4th and 5th exon.

Supplementary Figure 18 Out of the 6 breakpoints that occurred on this chromosome, four occurred within the genes including *ATXN1*, *CDK19*, *ARID1B*, and *SYNJ2*.

Supplementary Table 1 TIDDIT genome-wide structural variant calls for the proband. Yellow highlights denote the variants involved in this complex rearrangement.
