# Supplementary material for: Chromoanagenesis Event Underlies a de novo Pericentric and Multiple Paracentric Inversions in a Single Chromosome Causing Coffin–Siris Syndrome
Source: Front Genet. 2021 Aug 26;12:708348. doi: 10.3389/fgene.2021.708348 (PMC8427664; doi:10.3389/fgene.2021.708348)

Supplemental Figure 1:

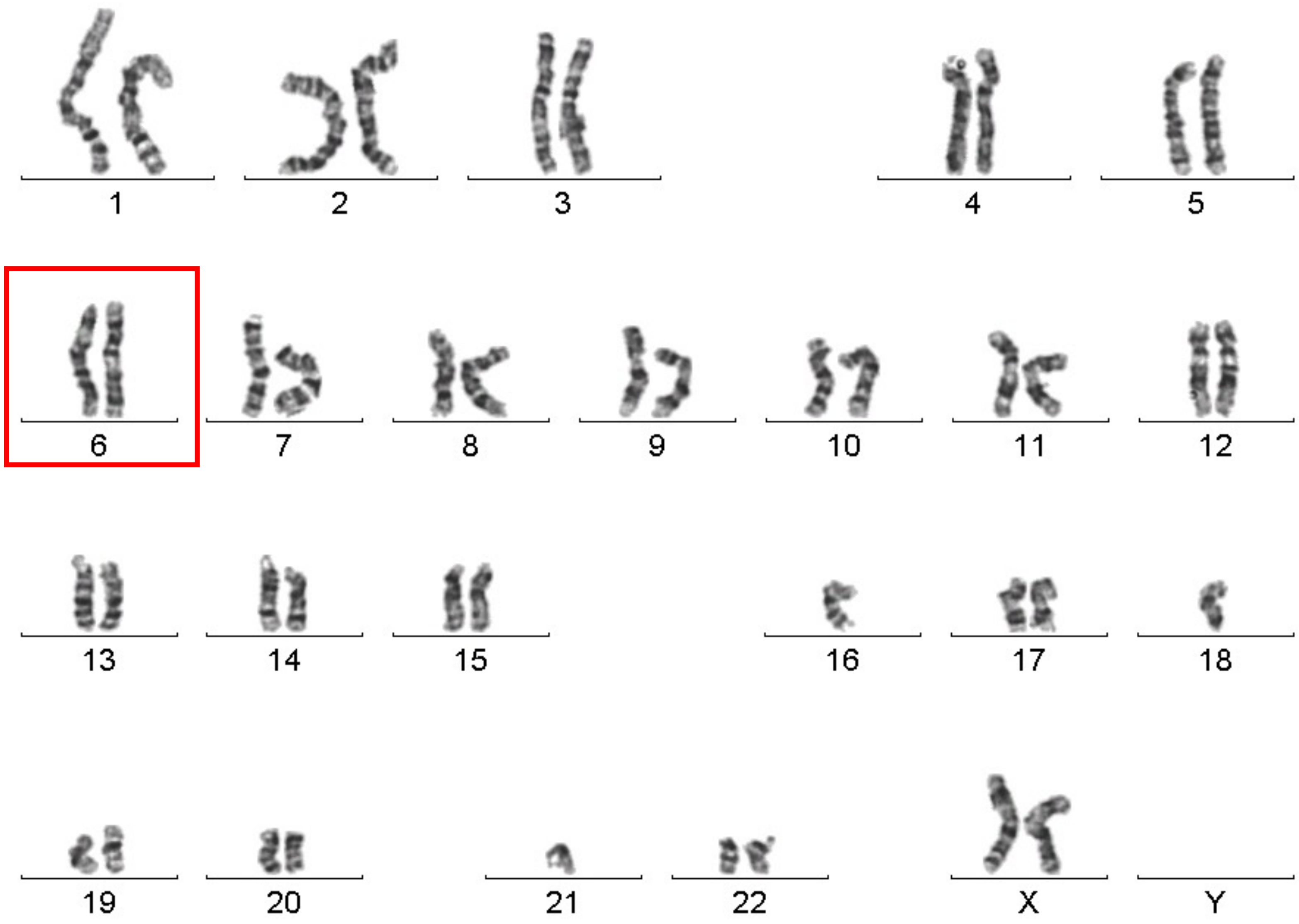

BAB12386

Supplemental Figure 2:

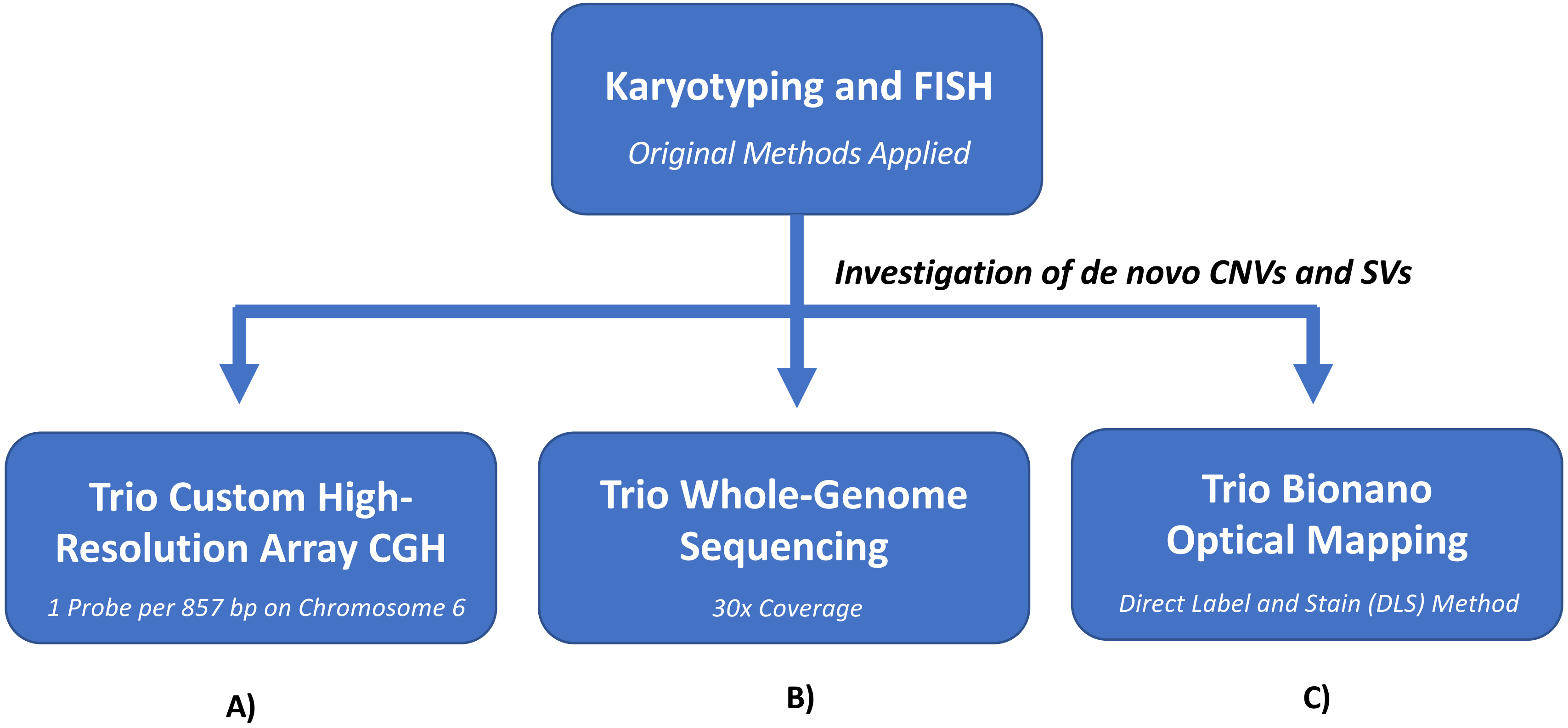

Supplemental Figure 3:

(A)

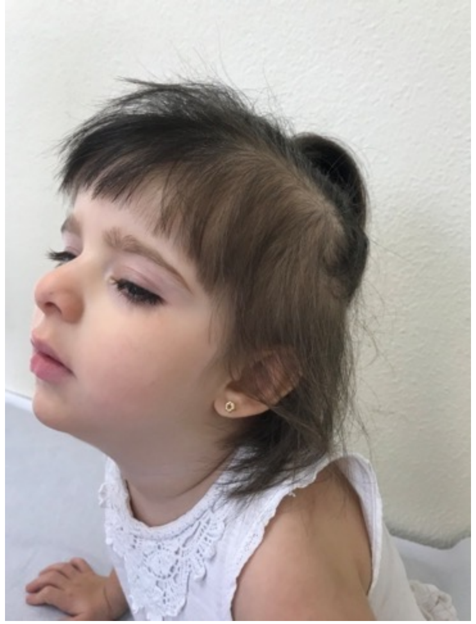

(B)

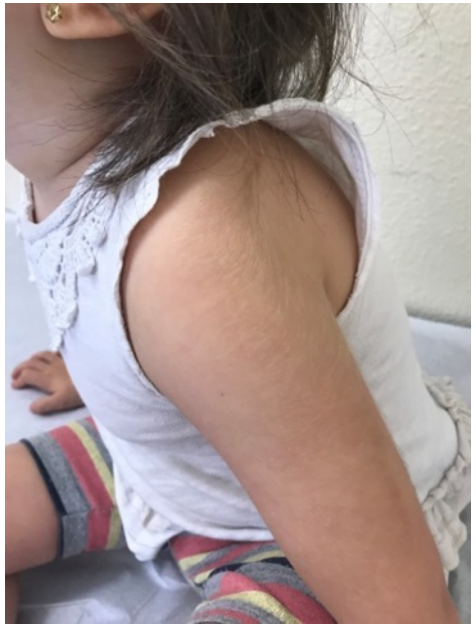

(C)

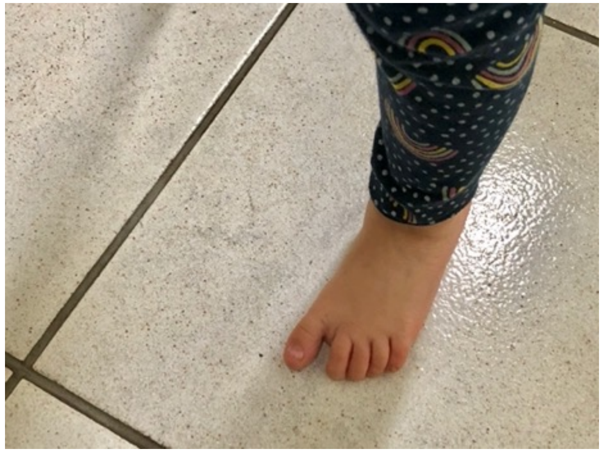

Supplemental Figure 4:

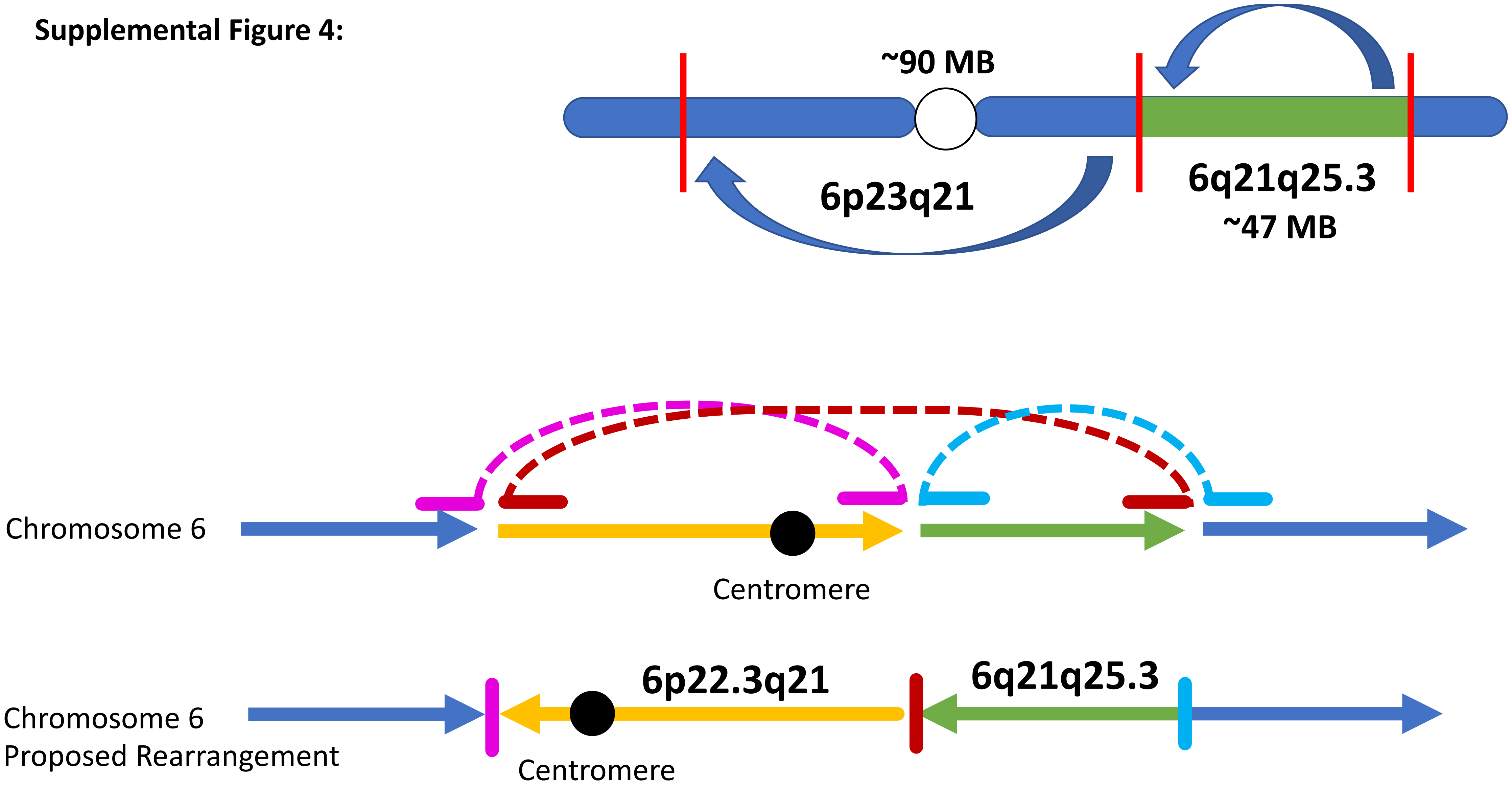

Supplemental Figure 5:

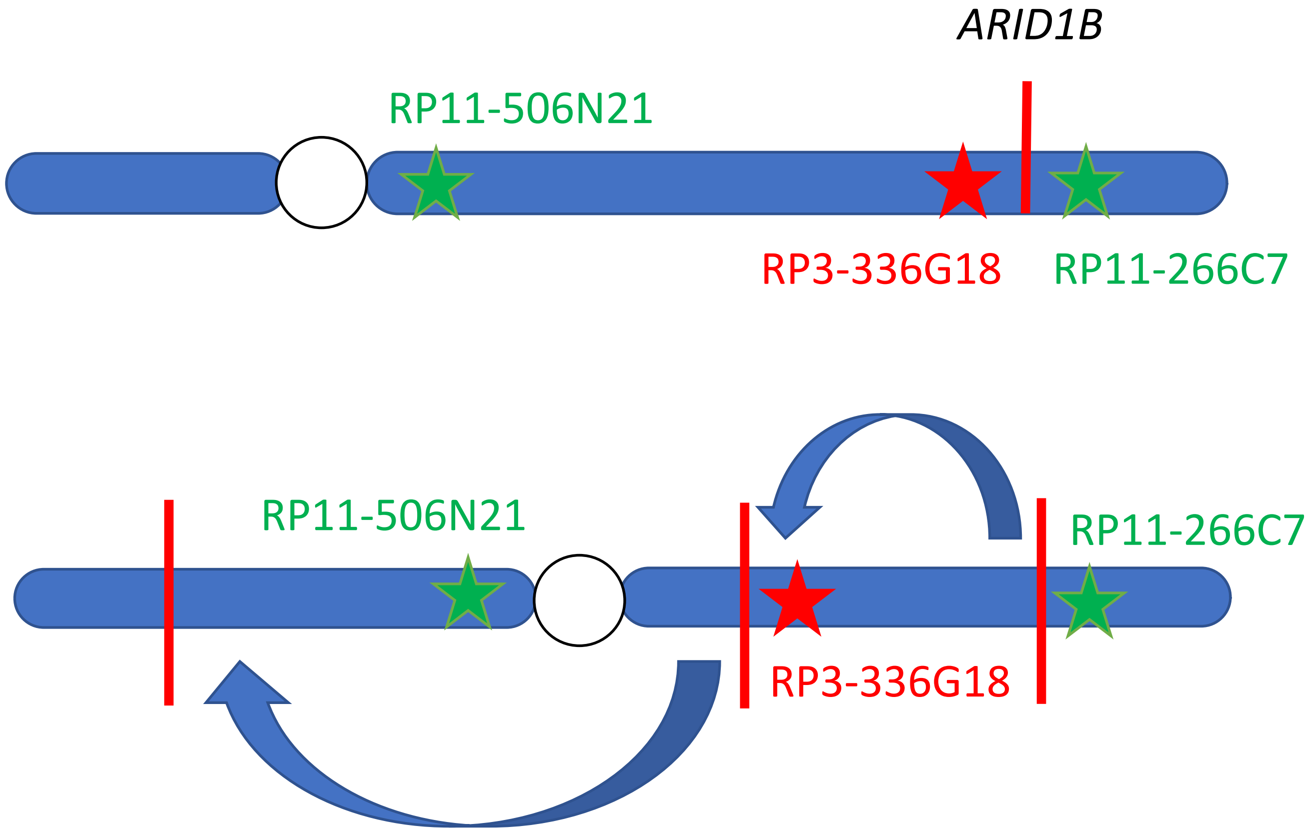

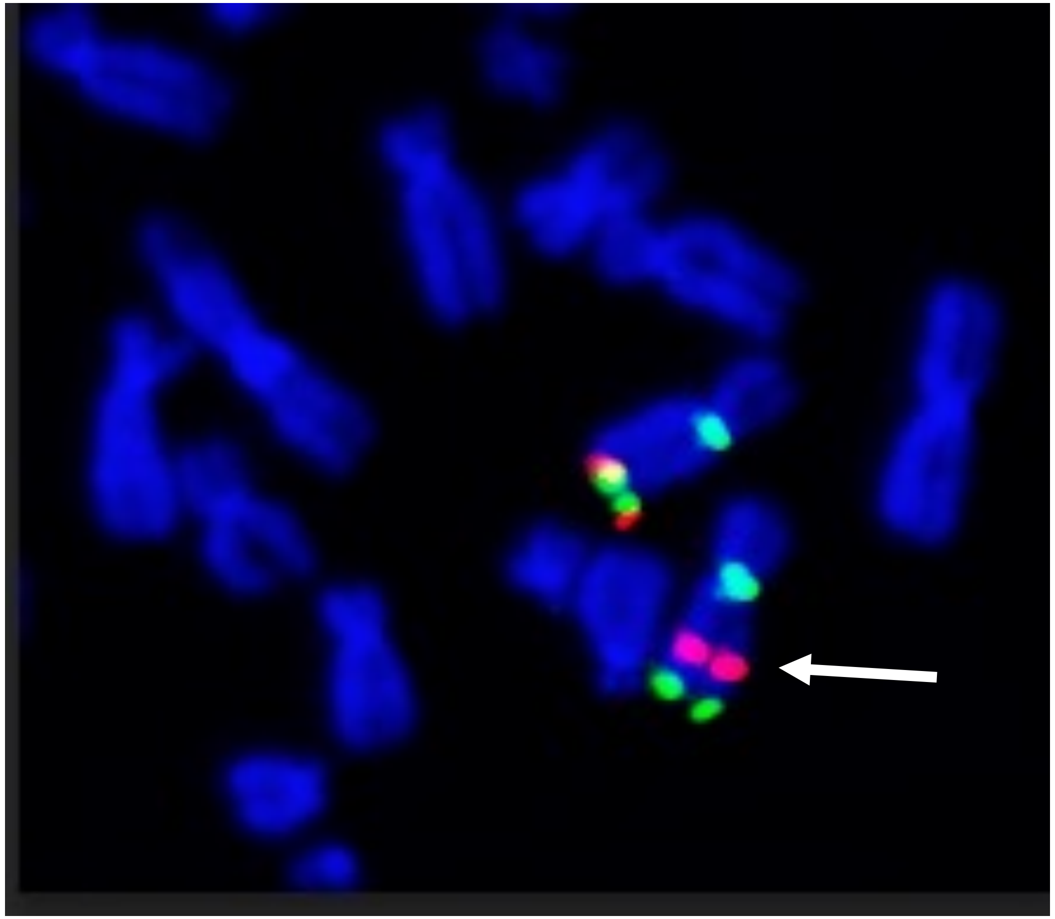

Supplemental Figure 6:

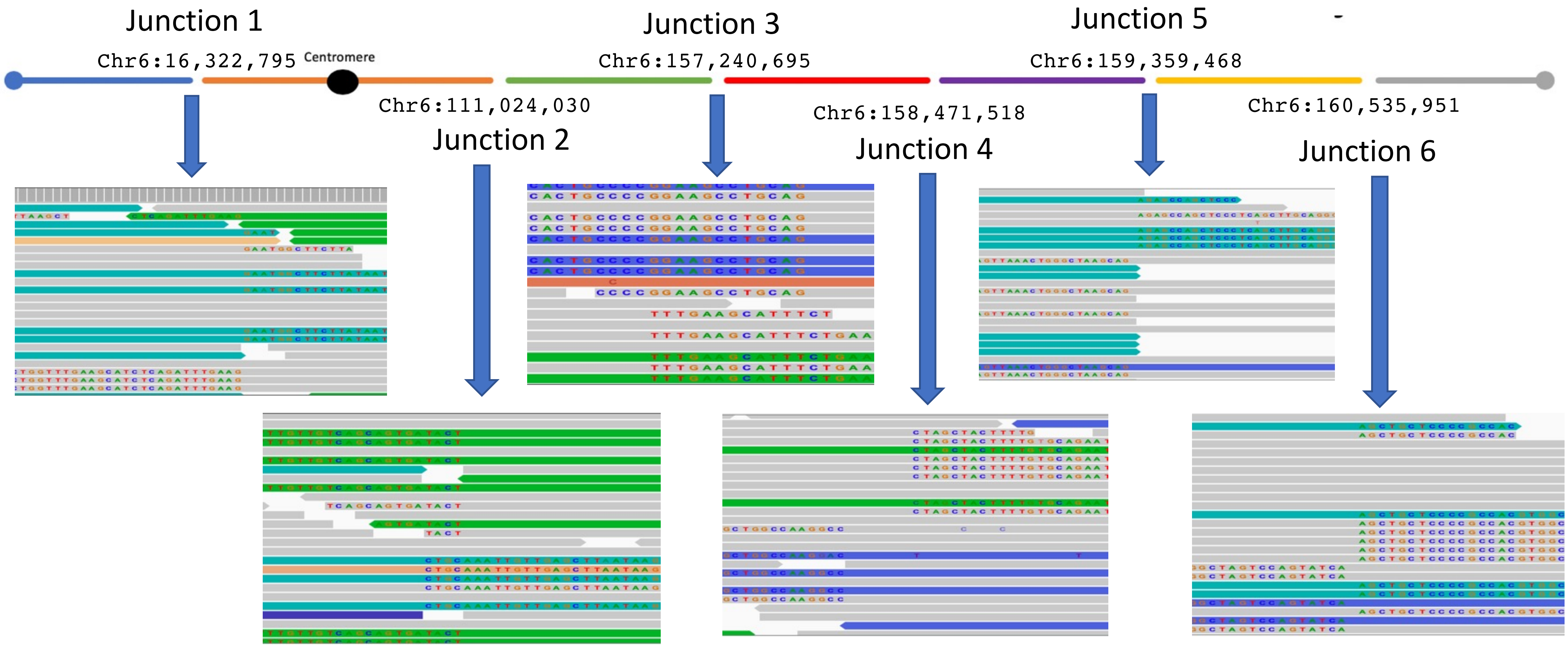

Supplemental Figure 7:

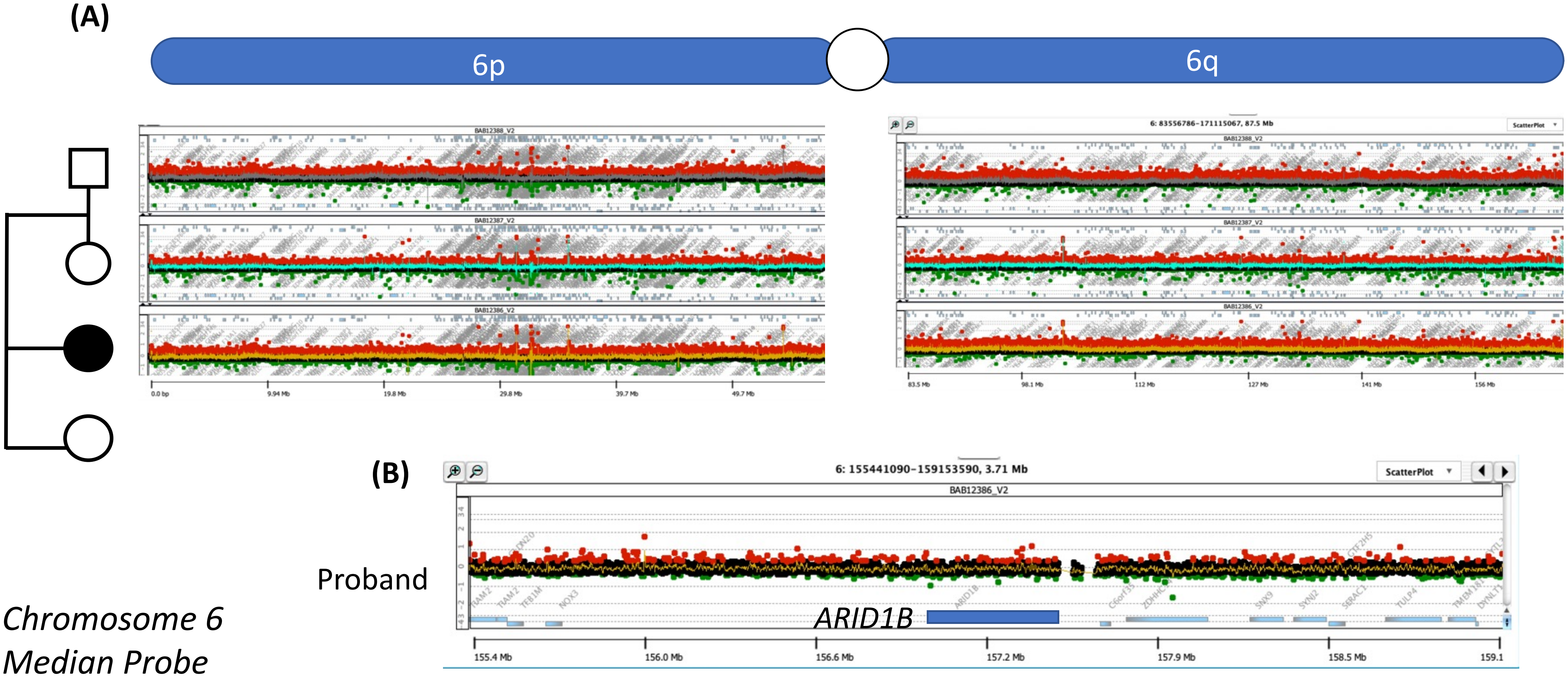

Chromosome 6
Median Probe
Spacing: 857 bp

Supplemental Figure 8:

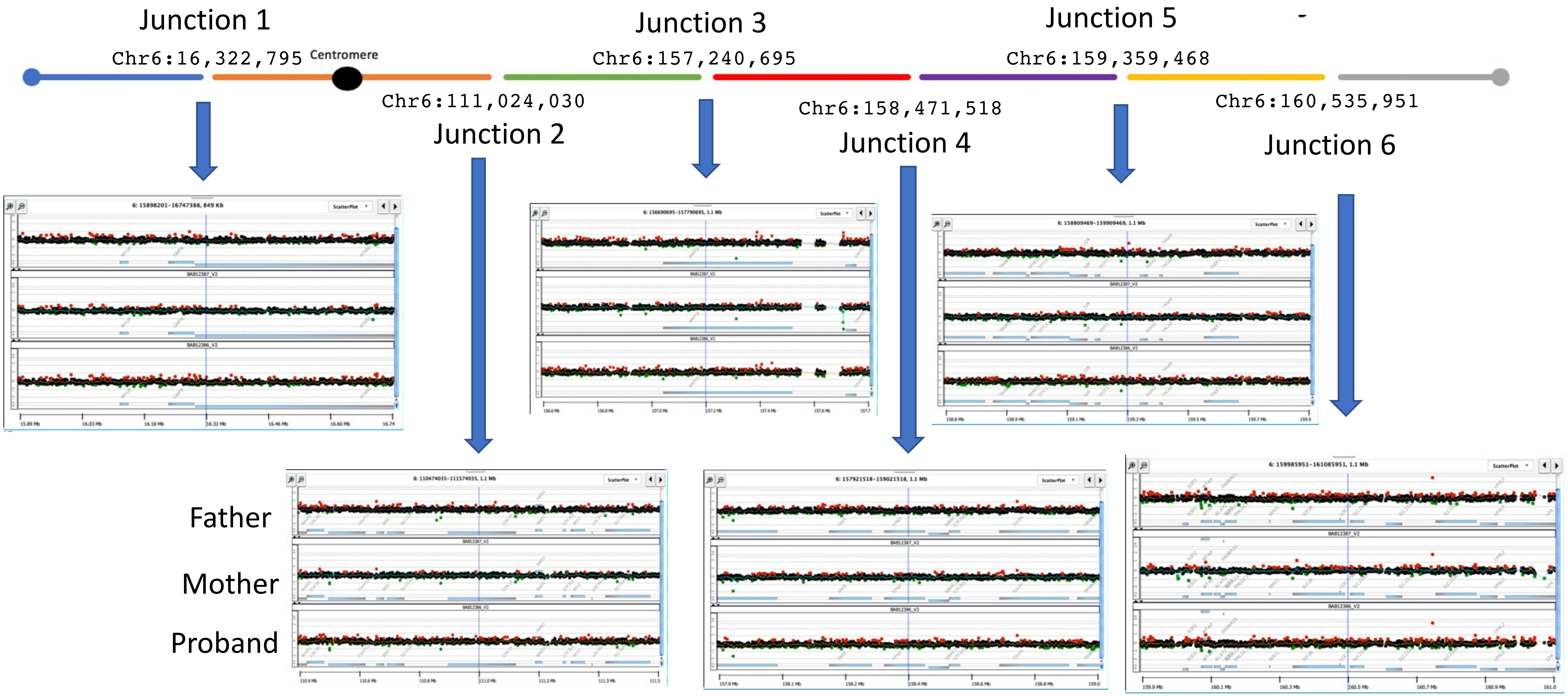

Supplemental Figure 9:

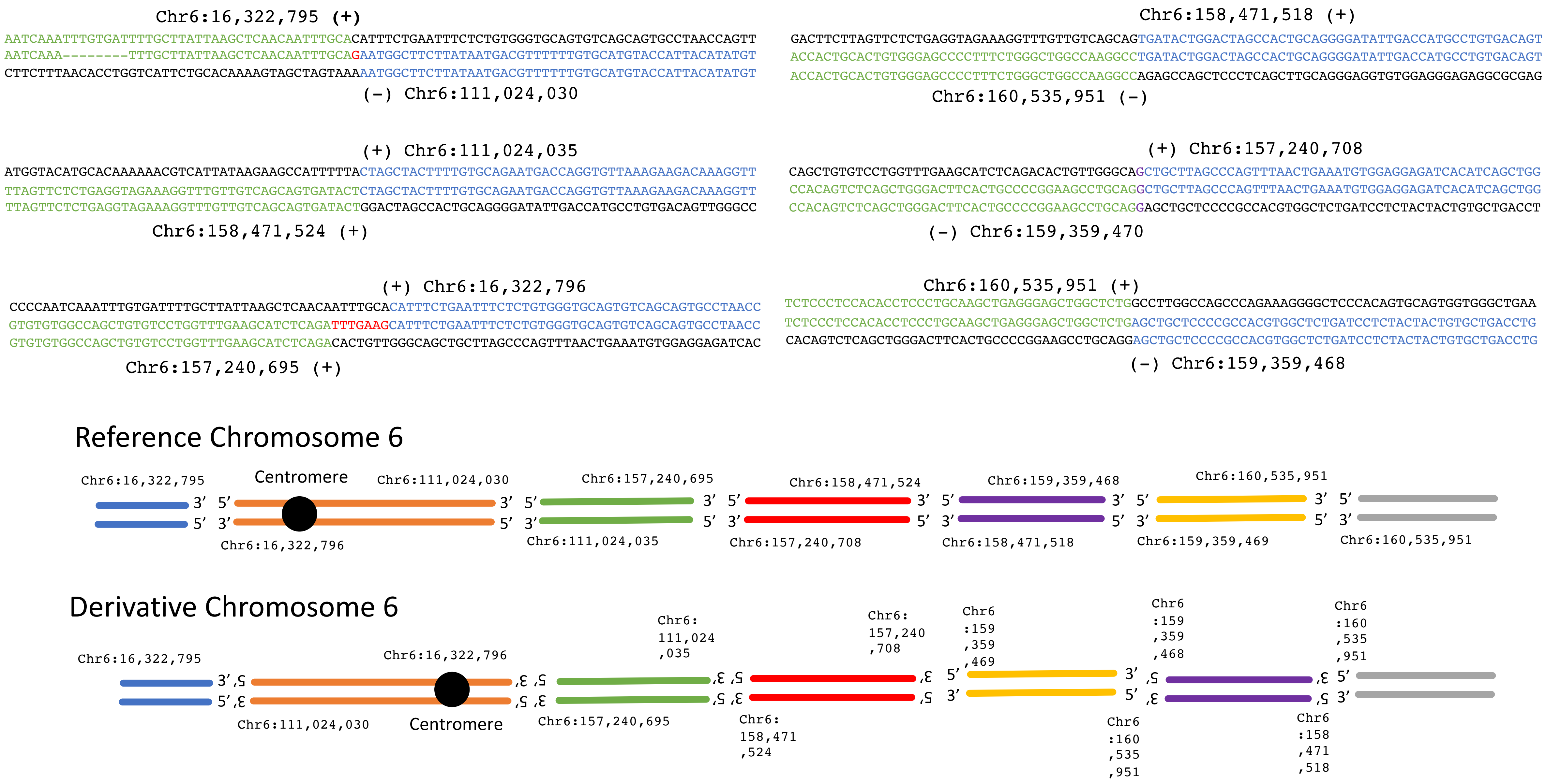

Supplemental Figure 10:

Junction 1

(A)

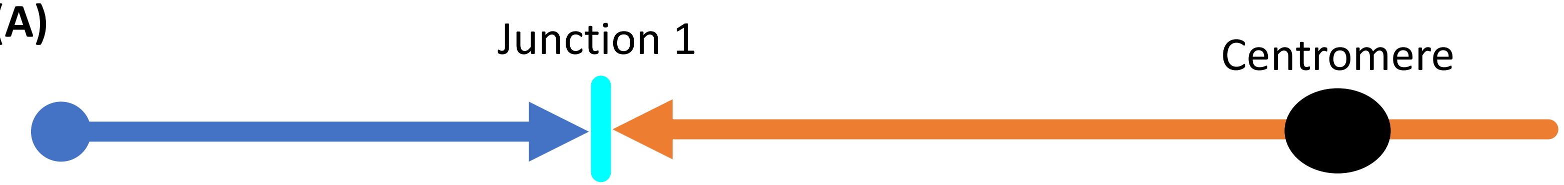

(B)

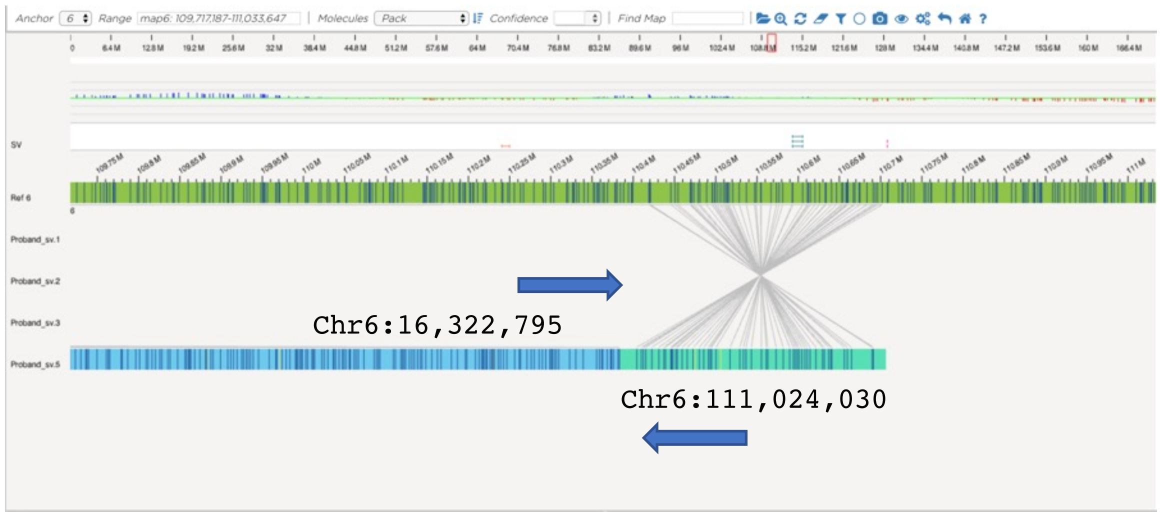

Supplemental Figure 11:

Junction 1

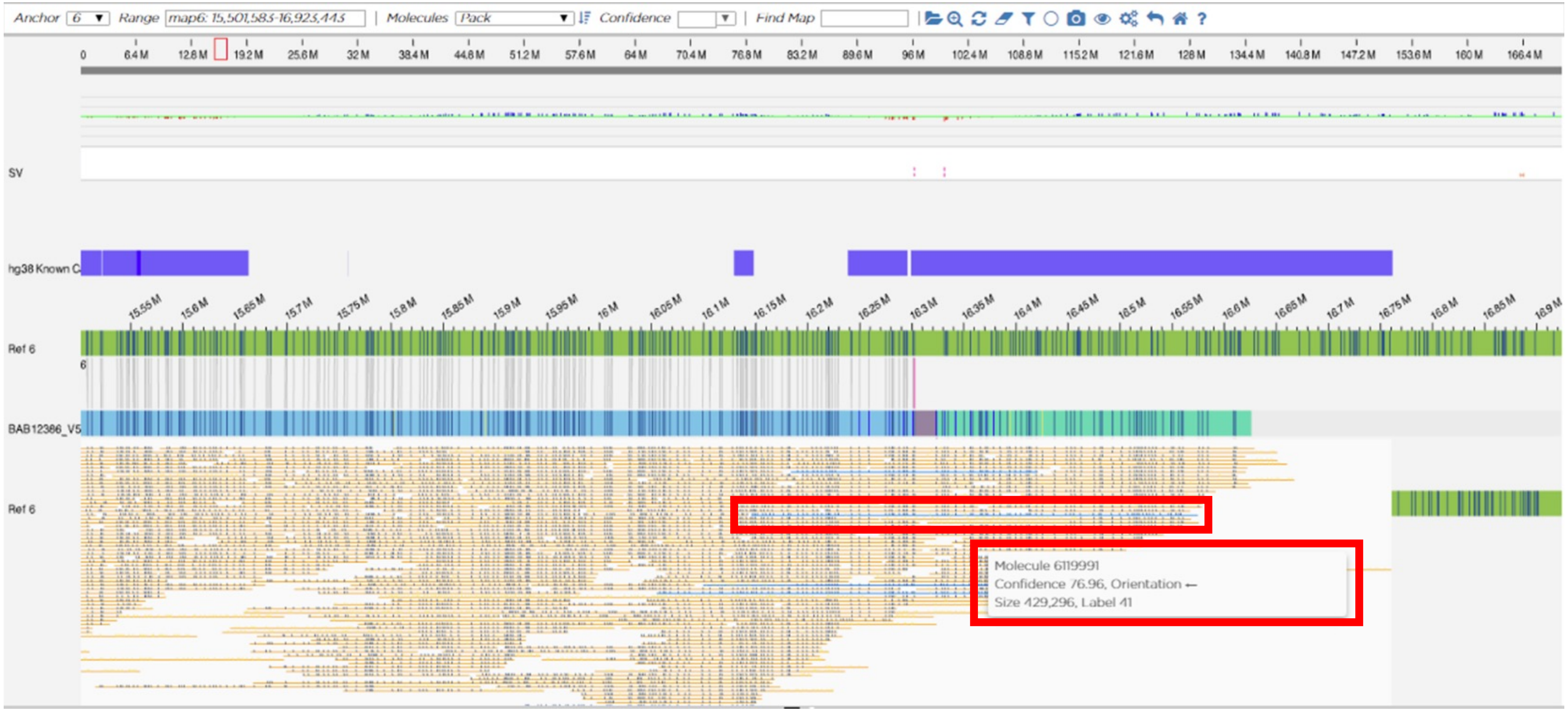

Supplemental Figure 12:

Junction 2

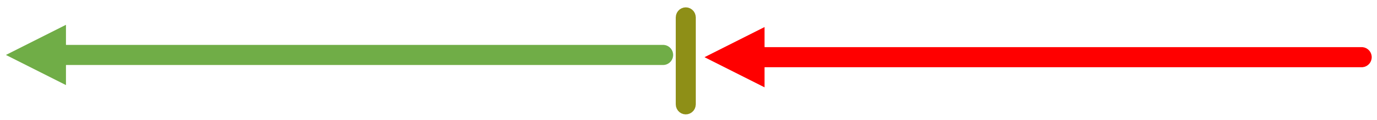

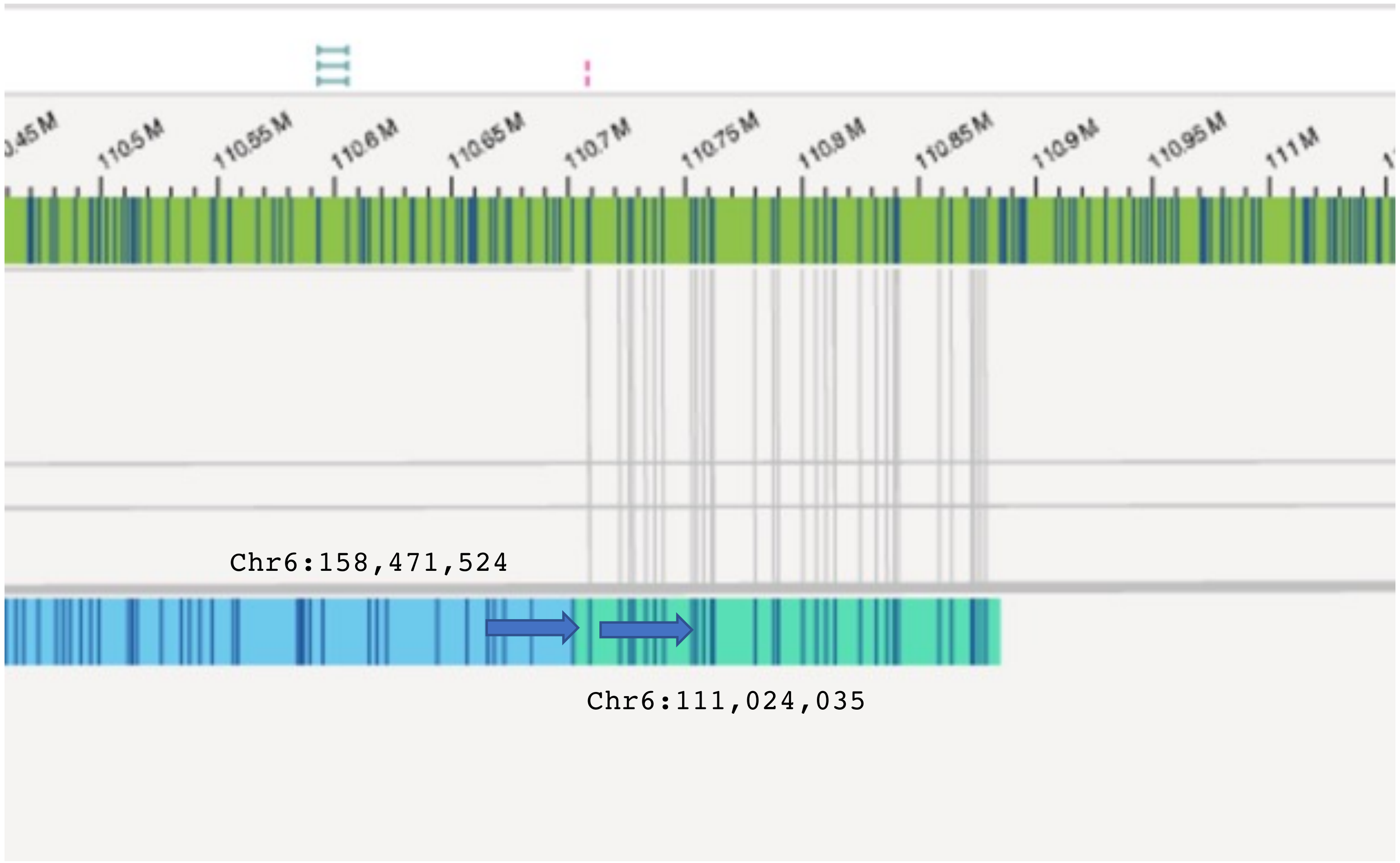

Supplemental Figure 13:

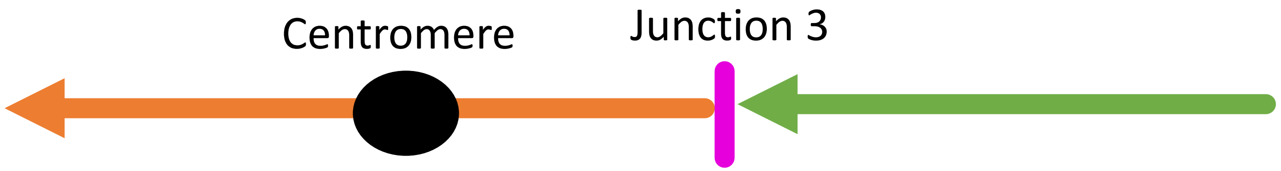

Junction 3

(A)

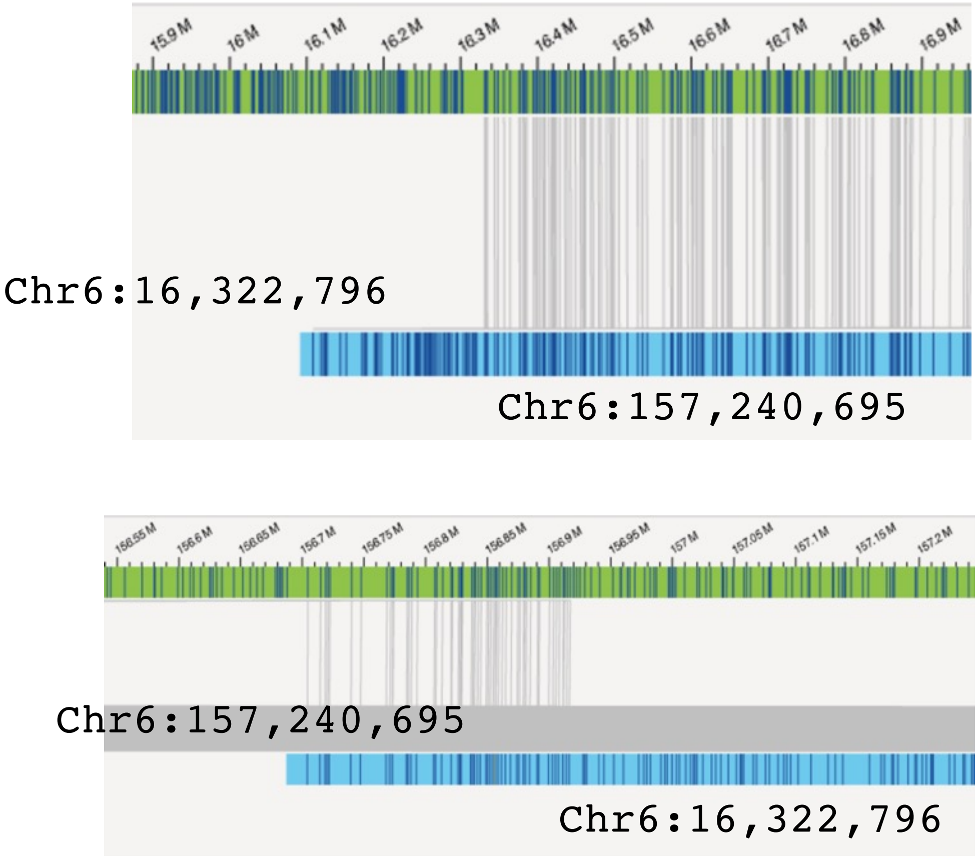

(B)

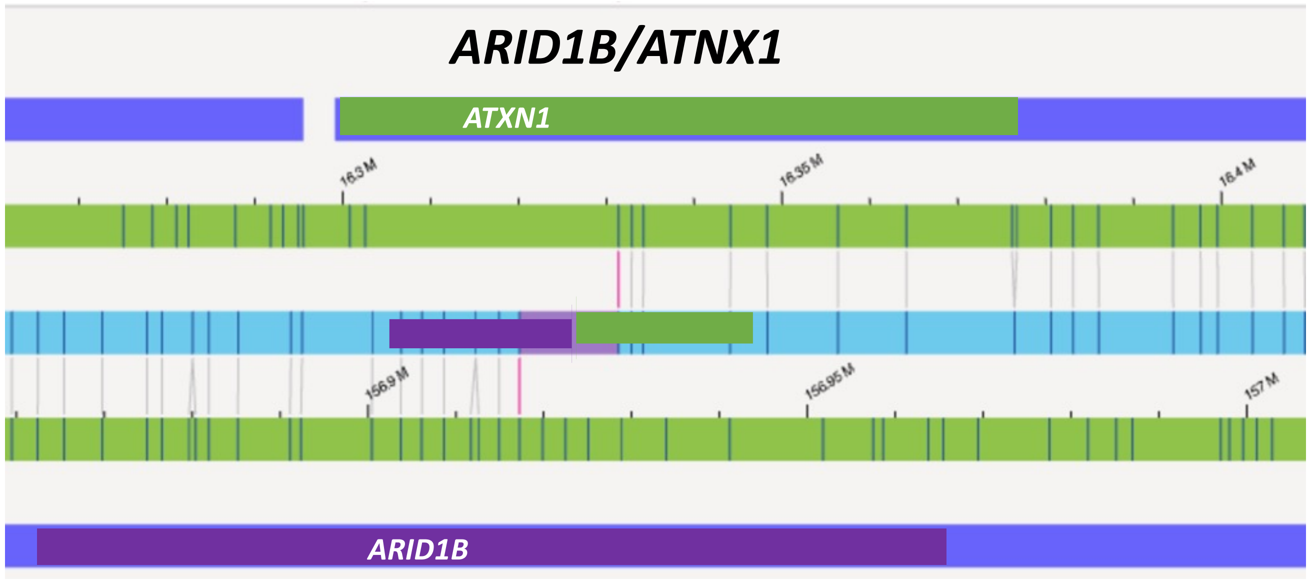

Supplemental Figure 14:

Junction 4

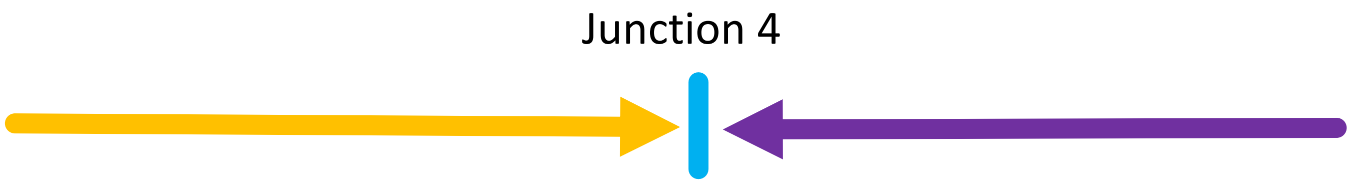

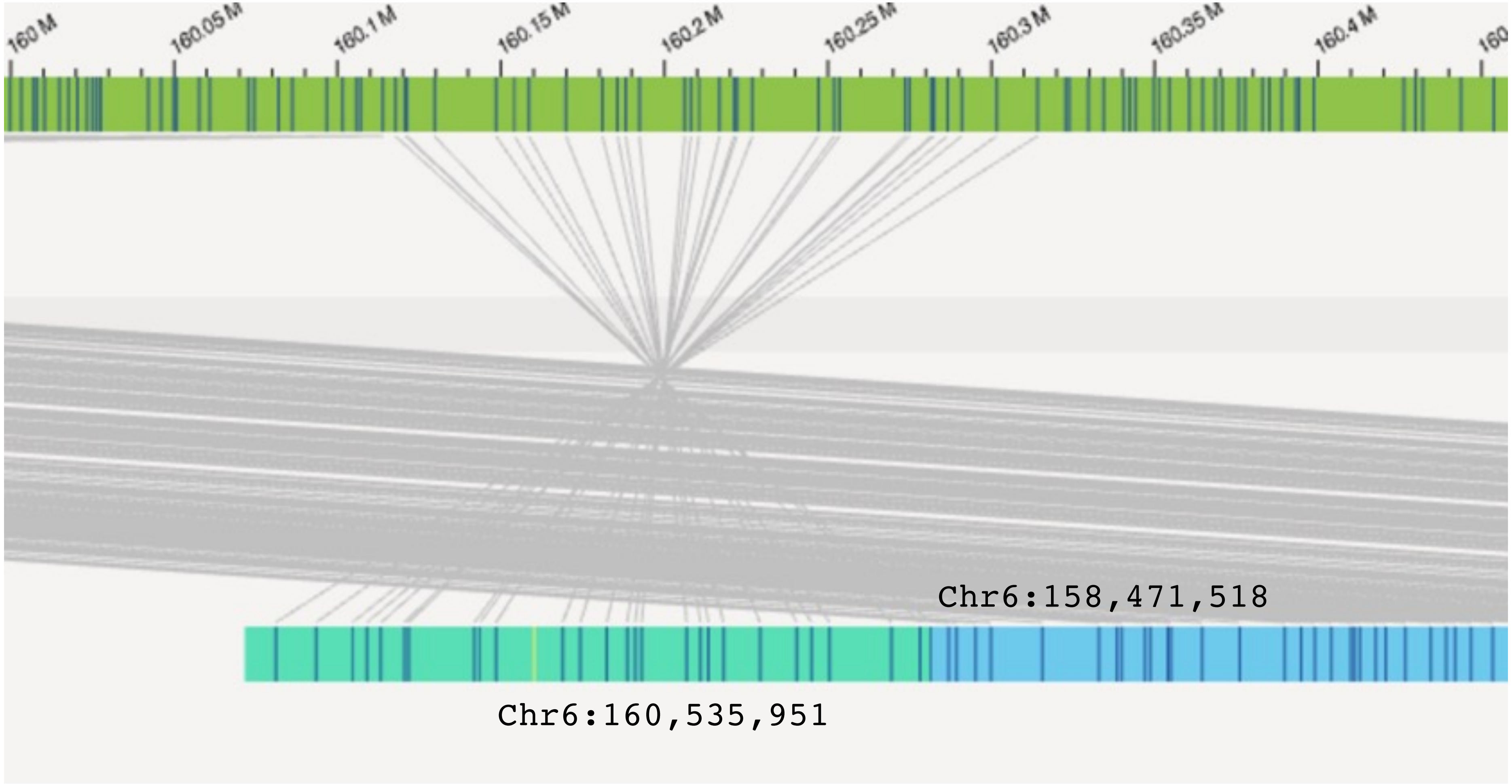

Supplemental Figure 15:

Junction 5

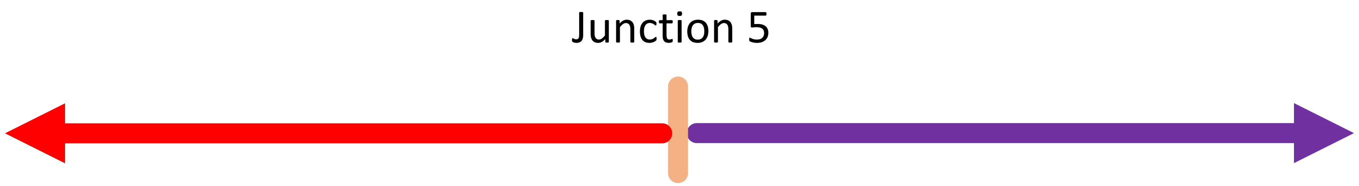

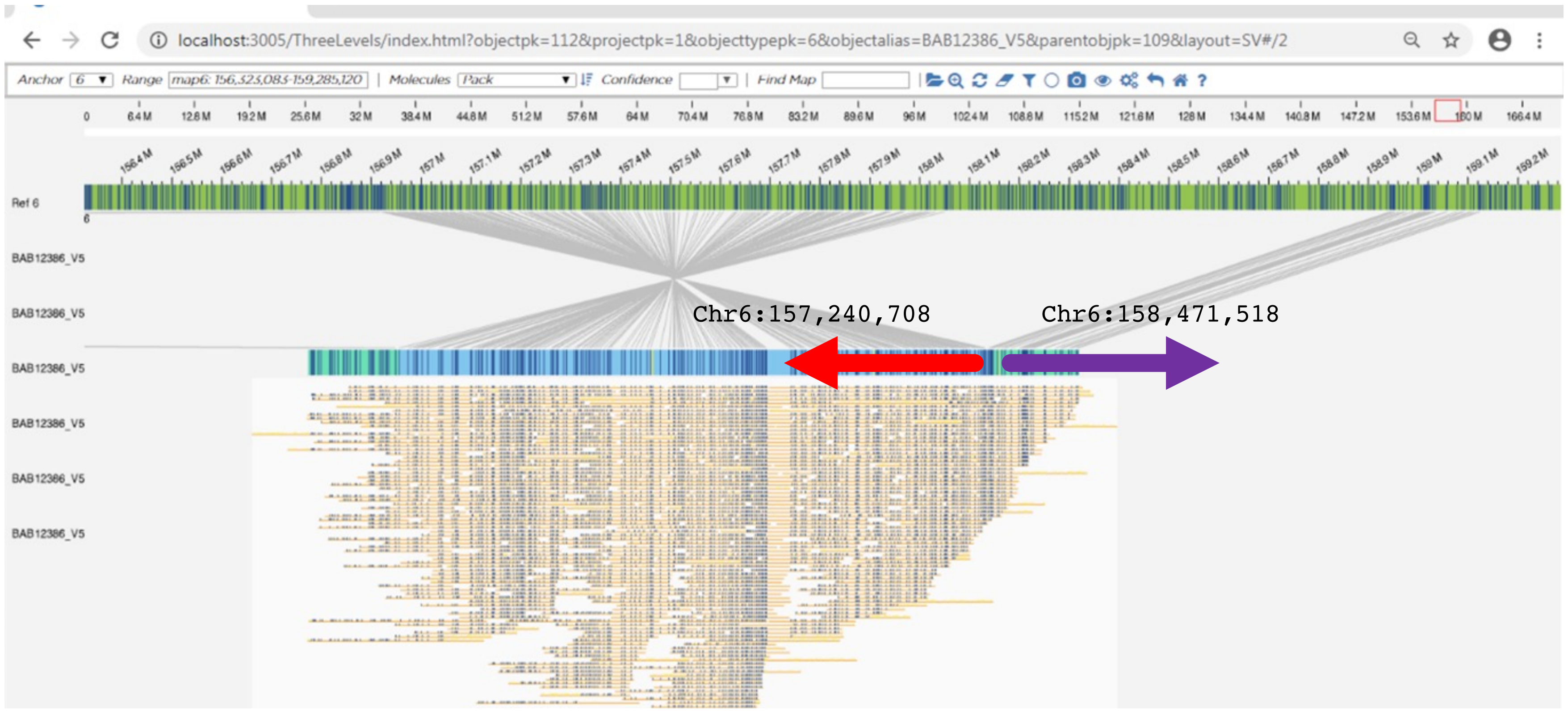

Supplemental Figure 16:

Junction 6

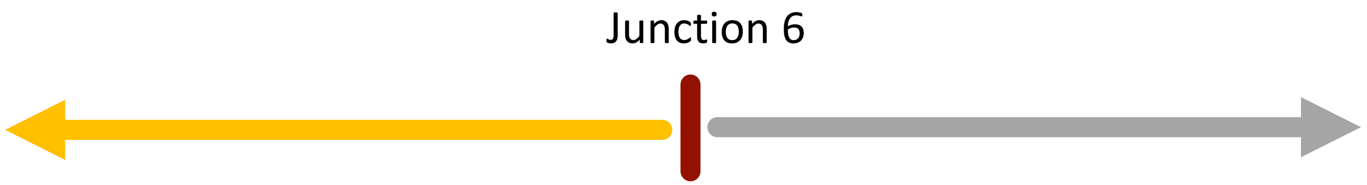

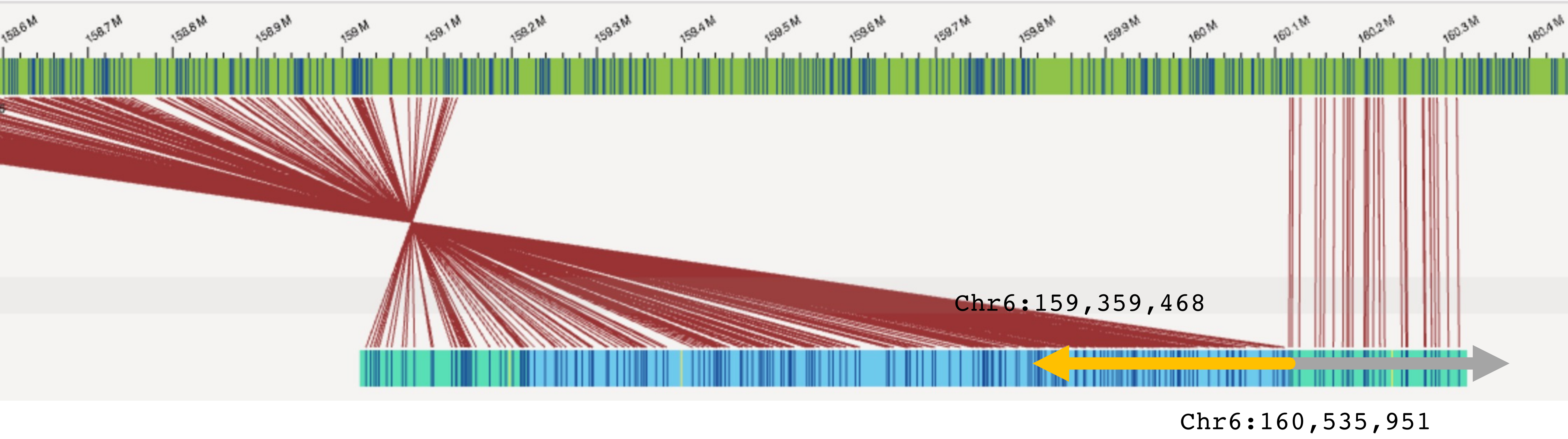

Supplemental Figure 17:

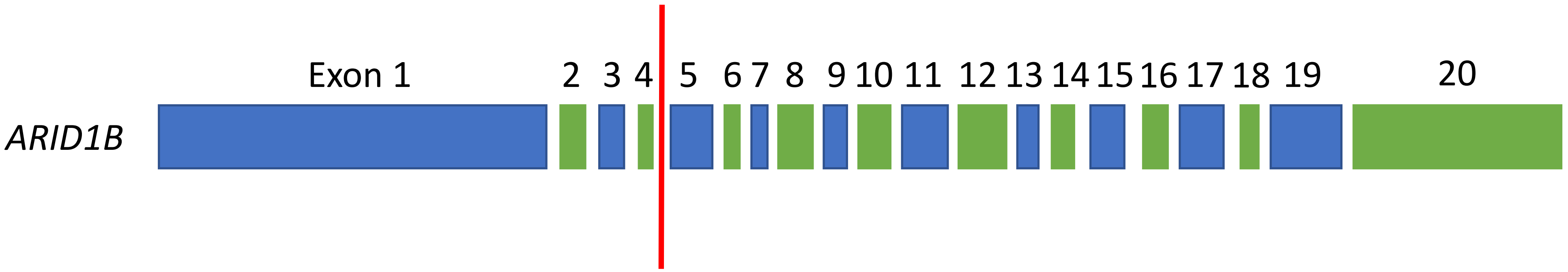

Supplemental Figure 18:

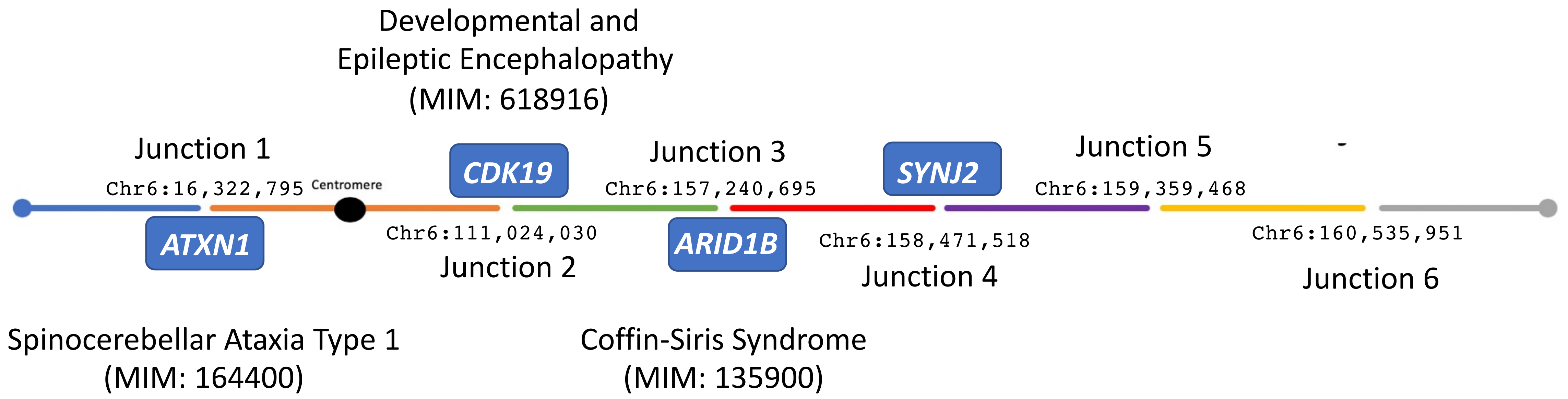

Supplement: Supplementary Figure 1 — Proband (BAB12386) full karyotype highlighting chromosome 6 within the red outlined box [46,XX, der(6)inv(6)(p23q21)inv(6)(q21q25.3)]. [file Data_Sheet_1.pdf]
