# Supplementary material for: Chromoanagenesis Event Underlies a de novo Pericentric and Multiple Paracentric Inversions in a Single Chromosome Causing Coffin–Siris Syndrome
Source: Front Genet. 2021 Aug 26;12:708348. doi: 10.3389/fgene.2021.708348 (PMC8427664; doi:10.3389/fgene.2021.708348)
Supplement: Supplementary Table 1 — TIDDIT genome-wide structural variant calls for the proband. Yellow highlights denote the variants involved in this complex rearrangement. [file Table_1.pdf]

Supplemental Table 1:

ChromosomeA	chromosomeB	PosA	PosB	Length	variant	frequency	ACC5958A2	signalPE	signalSR
1	2	21143225	235471765	#NUM!	BND	0	Het	7	0
1	8	24358623	9011515	#NUM!	BND	0	Het	5	0
1	10	36897237	101596736	#NUM!	BND	0	Het	13	0
1	10	53305647	9475025	#NUM!	BND	0	Het	9	15
1	1	145092950	145097082	4132	BND	0	Het	49	44
1	1	184402322	184403496	1174	DEL	0	Het	21	15
1	3	207580802	3158038	#NUM!	BND	0	Het	4	5
1	2	221363004	215201569	#NUM!	BND	0	Het	14	0
10	7	5640567	85173594	#NUM!	BND	0	Het	5	10
10	15	60492113	56882115	#NUM!	BND	0	Het	6	2
10	2	71610161	156897637	#NUM!	BND	0	Het	7	0
10	14	72814582	54361836	#NUM!	BND	0	Het	5	0
10	7	117966511	1367009	#NUM!	BND	0	Het	13	0
11	11	4305184	16910072	12604888	BND	0	Het	6	0
11	16	18149212	72729287	#NUM!	BND	0	Het	5	0
11	X	24623413	64670603	#NUM!	BND	0	Het	6	0
11	15	34347459	99553978	#NUM!	BND	0	Het	4	6
11	11	60754439	60758114	3675	BND	0	Het	10	8
11	21	95212800	21515121	#NUM!	BND	0	Het	5	0
11	21	106595882	32724753	#NUM!	BND	0	Het	9	4
11	5	110377879	152272015	#NUM!	BND	0	Het	9	0
12	2	7002491	11567906	#NUM!	BND	0	Het	5	0
12	X	23103219	172238	#NUM!	BND	0	Het	3	6
12	7	24253204	117449595	#NUM!	BND	0	Het	5	5
12	12	64655227	64656497	1270	DEL	0	Het	16	17
12	7	65177711	68280028	#NUM!	BND	0	Het	3	5
12	12	68161474	70483523	2322049	INV	0	Het	14	0
12	X	131328327	81772591	#NUM!	BND	0	Het	6	1
13	19	30610484	33709568	#NUM!	BND	0	Het	3	4
14	18	26878566	26463684	#NUM!	BND	0	Het	6	22
15	X	56962087	23798630	#NUM!	BND	0	Het	3	6
15	3	76372654	126648513	#NUM!	BND	0	Het	13	9
15	7	93861702	8372354	#NUM!	BND	0	Het	7	0
16	6	77564483	159782490	#NUM!	BND	0	Het	3	3
17	3	1180146	123042550	#NUM!	BND	0	Het	18	0

17	2	19531089	64738767	#NUM!	BND	0	Het	6	0
17	17	21556721	21558069	1348	BND	0	Het	7	18
17	X	31130225	126930315	#NUM!	BND	0	Het	9	0
18	8	827464	40459244	#NUM!	BND	0	Het	7	0
18	7	24307080	135180418	#NUM!	BND	0	Het	6	1
18	3	49216292	109836401	#NUM!	BND	0	Het	5	9
18	6	56769663	169465916	#NUM!	BND	0	Het	7	10
19	20	2382661	52032256	#NUM!	BND	0	Het	5	11
19	7	5796195	945062	#NUM!	BND	0	Het	3	11
19	19	47881279	47882367	1088	DEL	0	Het	13	17
2	2	47105663	110251338	63145675	BND	0	Het	4	11
2	9	203349105	115969625	#NUM!	BND	0	Het	4	10
2	4	234633964	151580011	#NUM!	BND	0	Het	16	0
20	4	35025083	8688897	#NUM!	BND	0	Het	4	11
3	5	13465475	15225838	#NUM!	BND	0	Het	6	6
4	7	15793162	130276187	#NUM!	BND	0	Het	8	0
4	4	78534142	78537404	3262	BND	0	Het	36	24
4	4	119748349	119750291	1942	DEL	0	Het	12	8
5	7	32091432	140190364	#NUM!	BND	0	Het	5	0
5	9	115139998	73589123	#NUM!	BND	0	Het	3	4
5	5	117613351	139859736	22246385	INV	0	Het	3	9
6	6	16322796	157240742	140917946	BND	0	Het	12	7
6	6	16322797	111024112	94701315	INV	0	Het	12	11
6	6	57284913	57289357	4444	BND	0	Het	30	18
6	6	111024035	158471590	47447555	BND	0	Het	20	16
6	6	157240708	159359469	2118761	INV	0	Het	12	15
6	6	158471518	160535951	2064433	INV	0	Het	12	4
6	6	159359471	160536020	1176549	INV	0	Het	17	12
7	8	47402886	22566935	#NUM!	BND	0	Het	4	8
7	9	70622362	16896100	#NUM!	BND	0	Het	5	0
7	9	90022458	135952694	#NUM!	BND	0	Het	6	4
7	X	101884400	46810708	#NUM!	BND	0	Hom	5	0
8	8	92099756	92103927	4171	BND	0	Het	18	16
8	8	92099766	92127319	27553	DUP	0	Het	8	8

Supplementary Table 1: TIDDIT genome-wide structural variant calls for the proband. Yellow highlights denote the variants involved in this complex rearrangement.
